# Jetting bubbles observed by x-ray holography at a free-electron laser: internal structure and the effect of non-axisymmetric boundary conditions

**DOI:** 10.1007/s00348-023-03759-9

**Published:** 2024-02-01

**Authors:** Juan M. Rosselló, Hannes P. Hoeppe, Max Koch, Christiane Lechner, Markus Osterhoff, Malte Vassholz, Johannes Hagemann, Johannes Möller, Markus Scholz, Ulrike Boesenberg, Jörg Hallmann, Chan Kim, Alexey Zozulya, Wei Lu, Roman Shayduk, Anders Madsen, Tim Salditt, Robert Mettin

**Affiliations:** 1 Drittes Physikalisches Institut, Georg-August-Universität Göttingen, 37077 Göttingen, Germany; 2 Faculty of Mechanical Engineering, University of Ljubljana, 1000 Ljubljana, Slovenia; 3 Institut für Röntgenphysik, Georg-August-Universität Göttingen, 37077 Göttingen, Germany; 4 Institute of Fluid Mechanics and Heat Transfer, TU Wien, 1060 Vienna, Austria; 5 CXNS - Center for X-ray and Nano Science, Deutsches Elektronen-Synchrotron DESY, 22607 Hamburg, Germany; 6 Helmholtz Imaging Platform, Deutsches Elektronen-Synchrotron, 22607 Hamburg, Germany; 7 European X-Ray Free-Electron Laser Facility, 22869 Schenefeld, Germany

## Abstract

In this work, we study the jetting dynamics of individual cavitation bubbles using x-ray holographic imaging and high-speed optical shadowgraphy. The bubbles are induced by a focused infrared laser pulse in water near the surface of a flat, circular glass plate, and later probed with ultrashort x-ray pulses produced by an x-ray free-electron laser (XFEL). The holographic imaging can reveal essential information of the bubble interior that would otherwise not be accessible in the optical regime due to obscuration or diffraction. The influence of asymmetric boundary conditions on the jet’s characteristics is analysed for cases where the axial symmetry is perturbed and curved liquid filaments can form inside the cavity. The x-ray images demonstrate that when oblique jets impact the rigid boundary, they produce a non-axisymmetric splash which grows from a moving stagnation point. Additionally, the images reveal the formation of complex gas/liquid structures inside the jetting bubbles that are invisible to standard optical microscopy. The experimental results are analysed with the assistance of full three-dimensional numerical simulations of the Navier–Stokes equations in their compressible formulation, which allow a deeper understanding of the distinctive features observed in the x-ray holographic images. In particular, the effects of varying the dimensionless stand-off distances measured from the initial bubble location to the surface of the solid plate and also to its nearest edge are addressed using both experiments and simulations. A relation between the jet tilting angle and the dimensionless bubble position asymmetry is derived. The present study provides new insights into bubble jetting and demonstrates the potential of x-ray holography for future investigations in this field.

**Supplementary Information:**

The online version contains supplementary material available at 10.1007/s00348-023-03759-9.

## Introduction

Oscillating and collapsing bubbles are main agents of erosion, surface cleaning, or other surface modifications by cavitation. A bubble collapse in a disturbed geometry will not proceed as under spherical symmetric conditions, and in many cases a liquid jet flow will emerge that penetrates the bubble interior gas phase during implosion (Lauterborn and Kurz 2010). The non-spherical collapse and jetting can have different origins, like neighbouring objects or phase boundaries, adjacent bubbles, a liquid flow, gravitational acceleration or the pressure gradient produced by a strong acoustic field (e.g. a shock wave) (Philipp and Lauterborn 1998; Lindau and Lauterborn 2003; Fujiwara et al. 2007; Supponen et al. 2015; Han et al. 2015; Sankin et al. 2005). Although many experimental and numerical studies have been dedicated to the investigation of the jetting phenomenon, it is sufficiently complex that many parts are still not fully understood. Reasons for experimental challenges for optical imaging comprise the small spatial scales and fast dynamics, but also the lack of a clear visualization of the gas cavity interior as a result of the reflections and scattering of the illuminating light at liquid–gas interfaces (Koch et al. 2021). The highly curved surface of the jetting bubbles deflect the light which actually goes into and through the cavity producing a significant distortion of the observed internal liquid filaments. These effects become increasingly relevant as the characteristic dimensions of the deformed cavity are reduced, and only a small fraction of the bubble is visible around its symmetry axis where the light rays impact the interface almost perpendicularly (Lauterborn and Bolle 1975; Rosselló et al. 2018). So far, the interior of a jetting bubble was only accessible for cases where the bubbles are produced in a transparent liquid, their expanded size is typically above 1 mm and a particularly intense multi-directional illumination was used (Lindau and Lauterborn 2003; Supponen et al. 2015; Koch et al. 2021). Even in those cases, the rather poor contrast of the images was not enough to clearly resolve the jet contour or only a part of it was discernible.

In the last decade, advances in high-speed x-ray imaging opened up an exciting range of possibilities as a novel experimental tool applied to research topics like: the propagation of shock waves in solids or liquids (Escauriza et al. 2020; Vassholz et al. 2021; Hagemann et al. 2021; Hodge et al. 2022; Vassholz et al. 2023) and their interaction with a gas cavity (Olbinado et al. 2017; Hodge et al. 2022; Montgomery 2023), ultrasound driven bubbles (Ehsani et al. 2021; Biasiori-Poulanges et al. 2023), fluid dynamics of cavitating flows in channels (Vabre et al. 2009; Lee et al. 2011; Morgan et al. 2013; Khlifa et al. 2017; Strucka et al. 2023) or in a bursting capillary (Vagovič et al. 2019) or bubble jetting (Hoeppe 2020; Bokman et al. 2023). X-ray imaging stands out from most optical techniques as one can probe complex gas–liquid interfacial structures, like the ones found in jetting bubbles, free of image distortions caused by refraction at boundaries (e.g. at curved interfaces). Furthermore, it can also be applied to optically opaque liquids.

The generally weak absorption of hard x-rays in matter allows to visualize the interior of the gas cavities by exploiting the phase shift induced by the sample, which, after a suitable distance of free-space propagation, leads to the formation of edge enhancement or Fresnel fringes when coherent X-ray beams are applied. The phase shift is proportional to the electron density projected along the optical path through the sample, which in turn depends on the mass density. This technique of x-ray phase-contrast holography is well developed in material- and life science providing spatial resolution from the micron-scale down to $20\,\hbox {nm}$ (Bartels et al. 2015). See Appendix A for a more extensive description of x-ray phase-contrast image formation. The advance of hard x-ray free-electron lasers (XFELs) now provides ultrashort x-ray pulses with sufficient numbers of photons to enable single-pulse imaging, and with that the investigation of fast hydrodynamic processes.

The erosion produced on a solid surface after the impact of a bubble jet is a well-documented phenomenon which attracted the attention of scientists and the industry for decades (Knapp et al. 1970; Karimi and Martin 1986; Philipp and Lauterborn 1998). In spite of that, the exact mechanisms leading to cavitation erosion are still under debate (Dular et al. 2019; Lechner et al. 2019; Koch et al. 2021; Reuter and Ohl 2021; Reuter et al. 2022; Dular and Ohl 2023), pointing to the localized high pressure produced upon jet impact on the surface as a possible cause for the observed damage. The impact of a bubble jet can also lead to piercing of biological tissue (Brujan et al. 2001; Ohl et al. 2006; Yuan et al. 2015) and soft gels (Rosselló and Ohl 2022), which is particularly important in medical applications like eye laser surgery or lithotripsy. The jet impact pressure is related with its speed, which in turn has a strong dependence on the dimensionless stand-off distance of the collapsing bubble to the surface, defined as $\gamma = d/R_{\mathrm{{max}}}$ (Lauterborn and Bolle 1975; Lauterborn et al. 2018), with *d* representing the distance from the bubble centre to the boundary and $R_{\mathrm{{max}}}$ the maximum radius of the bubble. The impact of the jet and the resultant splash are also relevant on processes that use cavitation for surface cleaning. The splashing produced after the jet has impacted the opposite bubble wall is formed when the spreading liquid of the jet meets the inflow that results from the proceeding bubble collapse (Lindau and Lauterborn 2003). This phenomenon has been described first in Blake et al. (1998); Tong et al. (1999). Recent literature terms it as the *Blake* splash (Lauterborn et al. 2018; Bokman et al. 2023).

In addition to the mechanical properties of the neighbouring surface, the jetting dynamics are also influenced by the specific boundary geometry. Some good examples of the latter can be found in cases where the bubble collapses near corners or mixed boundaries (Tagawa and Peters 2018; Kiyama et al. 2021; White et al. 2023; Li et al. 2023), sharp edges (Zhang et al. 2020), small platforms (Tomita et al. 2002; Koch et al. 2021; Kadivar et al. 2021; Lechner et al. 2023) or walls with an angle (Molefe and Peters 2019; Wang et al. 2020). Those studies demonstrate the effect of the boundary geometry on the jet direction, which is essentially determined by the relative stand-off distance from the bubble to the nearest flat surfaces. As the jetting bubble interior is not visible in most shadowgraphs, the jet direction becomes evident only by the liquid filament that pierces through the cavity on its re-expansion phase. In the cases with a reduced stand-off distance, e.g. $\gamma \lesssim 1$, the bubbles expand up to the point of touching the neighbouring surfaces, and as a consequence their shape becomes highly distorted. Due to the optical obscurity of the bubble interior, however, many aspects of the jetting dynamics in those cases remain unknown. Recently, Wang et al. (2020) and Li et al. (2023) employed a numerical *boundary integral method* (BIM) to study the jetting of bubbles near corners. Interestingly, their results suggest that the bubble jet can follow a curved trajectory as it penetrates the gas cavity (Wang et al. 2020). The limited number of works dealing with this kind of asymmetric cases can be explained by the technical challenges and the requirement of complex full 3D simulations, which demand a significant amount of computational resources.

In the present study we intend to clarify internal structures of collapsing laser-induced bubbles in the proximity of a solid boundary using time-resolved x-ray holographic imaging at the MID station of the European X-Ray Free-Electron Laser (EuXFEL) facility (Madsen et al. 2021). A comprehensive study of the dependence of bubble/jet dynamics on $\gamma$ for the axisymmetric case can be found in a recent numerical work by Lechner et al. (2020). Additionally, images of the interior of a jetting bubble under similar conditions were presented by Koch et al. (2021) and Bokman et al. (2023). In this ideal case, the solid boundary has close to infinite dimensions and the bubble volume is negligible compared to the volume of the liquid bulk. Here, we pay special attention to a non-ideal case found when a bubble collapses in “real life” situations, such as the mostly unexplored cases where the bubble collapses under non-axisymmetric boundary conditions (e.g. near the corner of a container or the border of a plate). These scenarios are expected to be more representative of generic and less-controlled environments, making their investigation essential for elucidating the mechanisms contributing to cavitation erosion.

## Experimental methods

**Fig. 1:**
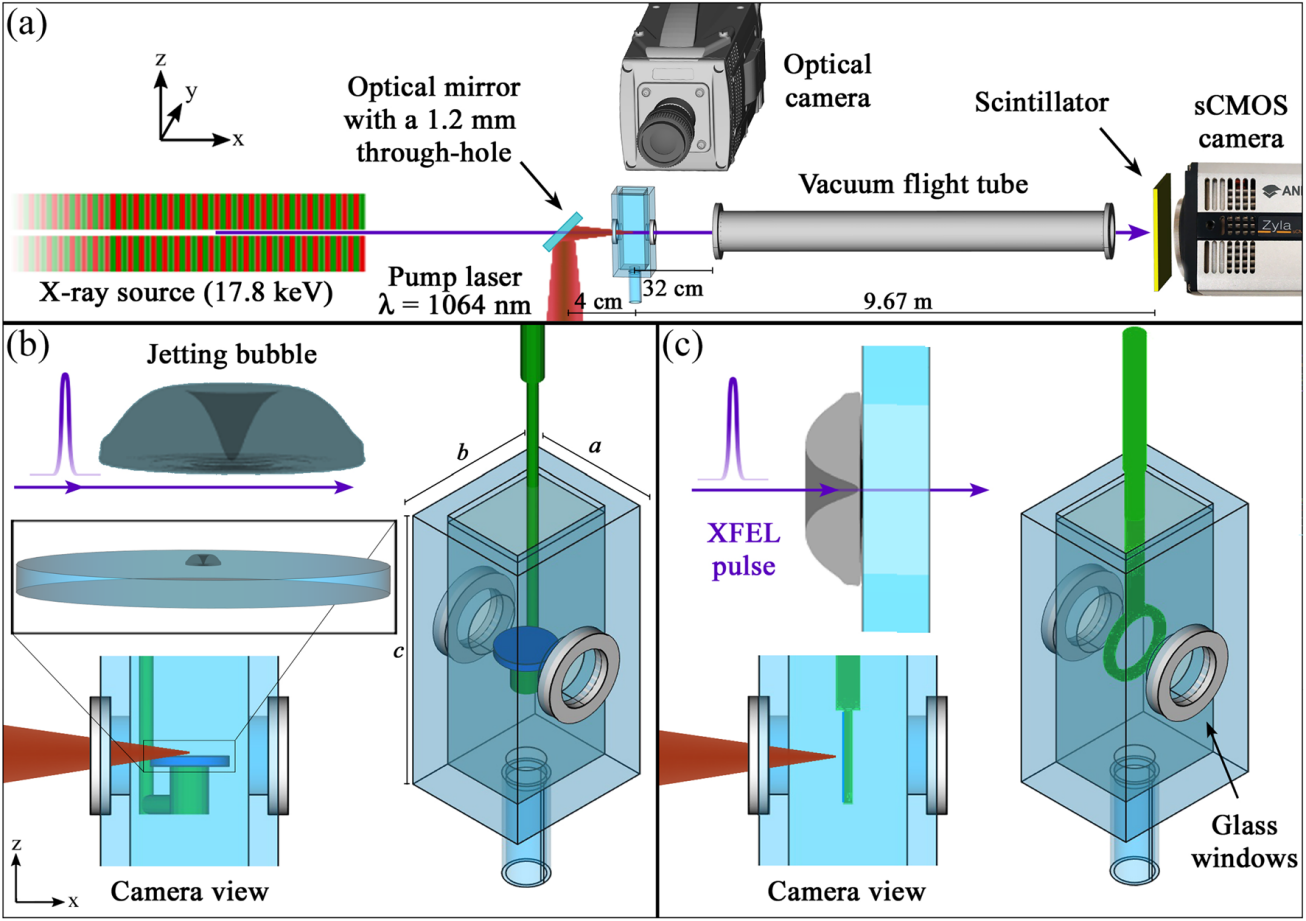
Experimental setup: Cavitation bubbles are seeded by an infrared pump laser close to a glass disc held inside an open cuvette filled with water. The jetting bubbles are probed by XFEL pulses while an optical camera observes from the side. **a** Schematic of the x-ray beam path in quasi-parallel beam illumination (not to scale). **b** The cuvette made from 3 mm acrylic plates has interior dimensions: $a=$12 mm, $b=$15 mm and $c=$36 mm. The glass plate (i.e. the blue disc) is placed in the horizontal plane, so both the x-ray and optical imaging feature a side view of the jetting bubble (perpendicular view). **c** Here, the glass surface is normal to the x-ray beam (parallel view)

In brief, the experiments consist of generating individual laser-induced cavitation bubbles in the proximity of a solid plate and then capture synchronous images of their jetting with an optical camera and x-ray holography from two orthogonal directions. An overview on the experimental setup at the Materials, Imaging and Dynamics (MID) instrument of the European XFEL (Madsen et al. 2021) employed in this study, is presented in Fig. 1a. The setup was operated at ambient conditions.

An infrared laser pulse (*Litron Nano L 200-10*, Q-switched Nd:YAG, $\lambda =$1064 nm, FWHM pulse duration of 6 ns, pulse energy of 17 mJ) is focused with a numerical aperture of $NA=0.2$ inside an acrylic cuvette filled with deionized water to produce the bubble. The collinear alignment of the laser-induced cavity and the x-ray light was ensured by reflecting the pump laser beam on a drilled mirror placed at 45^∘^ (hole diameter $\sim$ 1.2 mm) which allow the x-rays to reach the liquid filled cuvette as detailed in Fig. 1a. The distance between the x-ray detector and the sample, i.e. 9.67 m, is covered by an evacuated flight tube, which is tightly sealed with diamond and kapton windows.

The acrylic rectangular cuvette has interior dimensions: 12 mm $\times$ 15 mm $\times$ 36 mm and was equipped with a 5 mm cylindrical inlet at its bottom used to add or remove the liquid while holding the cuvette in a fixed position. Two circular windows with a diameter of 7 mm made from thin glass (i.e. $\sim$150 $\upmu$m of thickness) are placed at middle height of the cuvette to allow both the IR laser and the x-ray beam to reach the liquid without damaging the container walls and with a minimum energy absorption, see Fig. 1b and c.

In the experiments the boundary condition for the laser-induced bubble was given by a circular glass plate with a diameter of 8 mm that was initially placed with its centre aligned with the glass windows (and the x-ray beam) at the geometrical centre of the cuvette. This disc could be arranged in one of two orientations in order to visualize the bubble jetting from two different perspectives. In one case, described in Fig. 1b, a disc with a thickness of around 1 mm is held from below and with its surface normal oriented orthogonal to the x-ray beam, which allows observing the jetting bubbles from the side (i.e. perpendicular view). In the second case, a thinner glass plate (150 $\upmu$m) is placed with its normal parallel to the x-rays (and laser pulse) propagation direction (i.e. parallel view), as depicted in Fig. 1c. The cuvette and the glass plate holders (made from polyester, PLA) were mounted on independent XYZ-axis linear piezo stages (SLC 1730, Smaract, Germany), that allow a precise positioning of the disc with a spatial resolution of 4 nm.

The high-speed videos of the bubble dynamics were recorded with a *Photron Fastcam SA5* camera in combination with a long-distance microscope *Infinity K2* equipped with a *CF-3* lens. The camera frame rate was set to 75000 fps and the exposure time to 1 $\upmu$s in all the measurements. The high-intensity back-illumination needed for producing the shadowgraphs was provided by a *Sumita LS-M352* halogen lamp.

Ultrashort XFEL pulses with a duration below $100\,\hbox {fs}$ were delivered to the MID instrument (Madsen et al. 2021) with a 10 Hz repetition rate (single-bunch mode). XFEL pulses were generated at the SASE2 undulator line of the European XFEL, with an electron energy of 16.5 GeV. The photon energy was 17.8 keV, corresponding to a wavelength of 0.07 nm. The mean pulse energy during the beam time was $\sim$700 $\upmu$J. In this experiment no monochromator was used, so a SASE bandwidth of $\approx 10^{-3}$ is assumed (Geloni et al. 2010).

Single-pulse x-ray phase-contrast holography was performed on the cavitation bubbles in an in-line geometry with an unfocused, parallel x-ray beam. The diameter of the beam was chosen to be approximately 1 mm. The x-ray holograms were recorded with a scintillator-based fibre-coupled sCMOS camera (*Andor Zyla 5.5*) with a pixel size of 6.5 $\upmu$m, placed at a distance of $9.67\,\hbox {m}$ from the sample. In this context, the imaging regime with Fresnel number of $F_{pix}=0.062$ is a near-field holographic regime, where strong edge enhancement and Fresnel fringes are observed. Holographic contrast is formed by interference during free-space propagation of the XFEL pulse up to the detector. It is worth noting that contrast in the detected image is predominantly related to the object’s phase shift, while its absorption plays a minor role. We further illustrate the image formation and its processing in Appendix A. The x-ray pulses were synchronized with the CCD trigger in the optical camera with a precision of $\sim$ 100 ns. Due to the fast bubble dynamics, the bubble shape might suffer a noticeable change during the image acquisition time of 1 $\upmu$s set in the optical camera; consequently, the events shown in the optical images might slightly differ from the x-ray holographic images, which are taken in a much shorter temporal window (i.e. $100\,\hbox {fs}$).

The timing and synchronization scheme of this experiment is described in detail in the work of Osterhoff et al. (2021). In short, a pump-probe experiment is performed, where a new cavitation bubble is seeded by the optical laser for every XFEL probe pulse, delivered with 10 Hz repetition rate. Additionally, an optical high-speed video sequence is recorded for each event. All devices were triggered using electronic delays relative to a master XFEL trigger.

## Numerical methods

The experimental x-ray holographic images were compared to numerical simulations computed with the *Finite Volume method* (Ferziger and Perić 1997). The computational fluid dynamics solver is built from the segregated, pressure based, two-phase compressibleInterFoam solver (Miller et al. 2013) within the OpenFoam-package (Jasak 1996; OpenFOAM-v2006 2020), precisely the foam-extend fork. The numerical simulations were performed using a full three-dimensional mesh of the cuvette used in the experiments, which allowed us to study the physics behind the x-ray images of the jetting of bubbles, as well as interpolate and extrapolate cases not covered by the experiments.

### Definition of dimensionless parameters

**Fig. 2:**
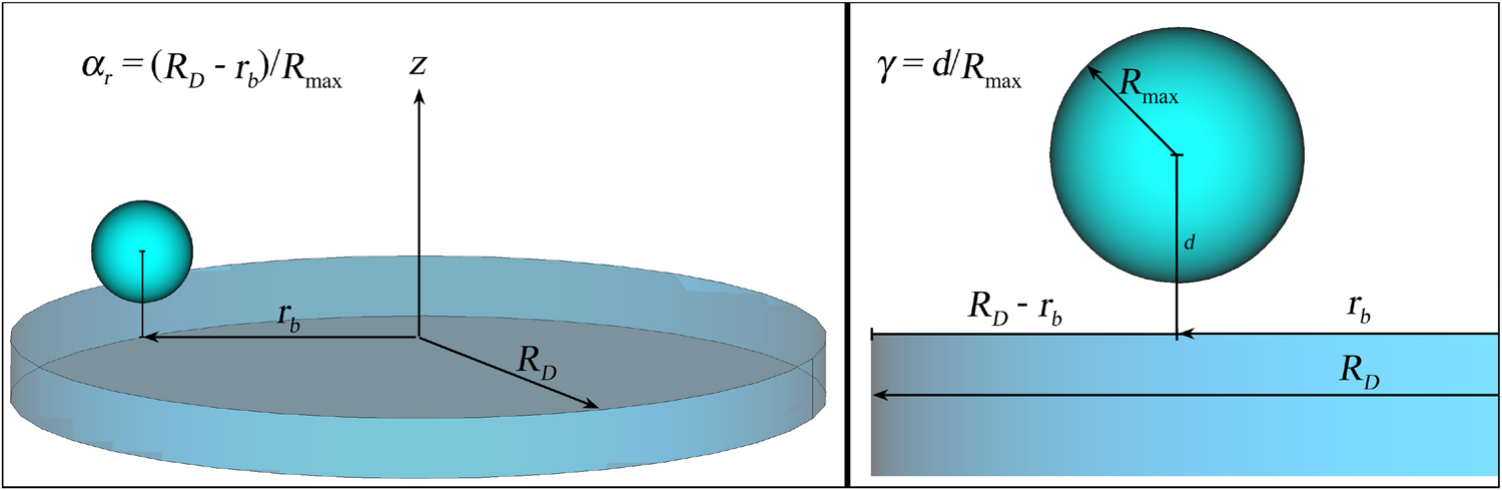
Distances and dimensionless parameters used to characterize the bubble dynamics

In the following, the bubbles are characterized by two dimensionless numbers computed from the geometry as illustrated in Fig. 2: the previously mentioned normalized stand-off distance of the bubble centre to the solid surface $\gamma = d/R_{\mathrm{{max}}}$, and a normalized stand-off distance of the bubble centre to the border of the disc, defined as $\alpha _r=(R_D-r_b)/R_{\mathrm{{max}}}$. Here, $r_b$ denotes the radial bubble seeding position measured from the centre of the circular glass plate, and $R_D$ is the radius of the disc. In this scaling, the bubble is located just above the edge for $\alpha _r=0$, and a bubble at the symmetry axis would mean $\alpha _r=R_D/R_{\mathrm{{max}}}$. In the experiments, the maximum radius $R_{\mathrm{{max}}}$ was measured by fitting a circle in the upper half of the optical images of the bubbles (i.e. located away from the surface). In the simulations, a similar method was employed for the sake of consistency. It can be assumed that the absolute size of the disc does not have a significant impact on the jetting dynamics as long as $R_D \gg R_{\mathrm{{max}}}$. Consequently, the use of a dimensionless parameter like $\alpha _r$ that does not account for the exact disc size is justified.

The temporal evolution of the cavities is compared by computing a prolongation factor in the collapse time, defined as the time measured from the laser shot ($t=0$) to the moment where the gas phase reaches its minimum volume ($t(V_{\mathrm{{min}}})$), normalized by two times the Rayleigh collapse time of an empty spherical bubble of same maximum radius, $(2\,T_c(R_{\mathrm{{max}}}) = 1.829 \,R_{\mathrm{{max}}}\sqrt{\rho _0/p_\infty }$, where $\rho _0$ and $p_\infty$ denote the density and pressure of the liquid. Additionally, we defined a normalized maximum volume, $V^*_{\mathrm{{max}}}$, as the ratio of the bubble gas volume and the volume of an unbounded cavity produced under similar conditions.

### Bubble model, CFD solution method, mesh and initial conditions

The numerical model and solution method is briefly sketched here and has been developed and validated with experimental data over the past decade (e.g. see Refs. Koch et al. 2016; Lechner et al. 2017, 2019, 2020; Koch et al. 2021). The bubble contains a small amount of non-condensable gas, the vapour pressure is neglected. While hydrodynamically nucleated cavitation bubbles are composed of mainly vapour, laser-induced bubbles like the ones produced in this experiment contain mostly non-condensable gases. These gases appear due to molecular dissociation-recombination reactions from the plasma. Presumably, the main gas is hydrogen (Maatz et al. 2000), but probably also oxygen and other recombination products exist (e.g. see Table 1 in Schanz et al. (2012)). Therefore, the neglecting of vapour is justified for the cases presented. The non-condensable gases are summarized to an ideal gas with polytropic exponent of 1.4 in the model. The model further assumes a cold liquid, i.e. a liquid far from its boiling point, with the following properties. The gas and also the liquid are taken as compressible in order to allow for a realistic modelling of sound and shock wave emissions and the respective losses during collapse. Viscosity is included in both fluids. Thermodynamic effects and mass exchange through the bubble interface are neglected. Gravitational effects can be omitted due to the small size and fast dynamics of the bubble.

The equations of motion of the two-phase flow are formulated in the “one-fluid” approach, i.e. with one density field $\rho (\vec x, t)$, one velocity field $\vec U(\vec x, t)$, and one pressure field $p(\vec x,t)$, satisfying the Navier–Stokes equation and the continuity equation for compressible fluids. Although surface tension plays a minor role for the cases considered here, it is included in the momentum equation via a volume force term. The surface tension coefficient $\sigma$ of water is set to $\sigma = 0.0725\mathrm {kg\,m}^{-2}$. In order to distinguish between liquid (*l*) and gas (*g*), a volume fraction field $\alpha (\vec x,t)$ is introduced with $\alpha = 1$ in the liquid phase, $\alpha =0$ in the gas phase. The position of the interface is then given implicitly by the transition of $\alpha$ from 1 to 0. The dynamic viscosities $\mu _l$ of the liquid and $\mu _g$ of the gas are taken to be constant ($\mu _l = 1.002 \times 10^{-3}\,{\textrm{kg}\ {\mathrm s}^{-1}\,{\mathrm m}^{-1}}$, $\mu _g = 1.7 \times 10^{-5}\,{\textrm{kg}\ {\mathrm s}^{-1}\,{\mathrm m}^{-1}}$).

The equations of motion are closed by the equations of state for the gas and the liquid. For the gas in the bubble, the change of state is assumed to be adiabatic, i.e. $\rho _g(p)/p^{1/\gamma _g} = const.,$ with $\gamma _g = 1.4$ the ratio of the specific heats of the gas (air). For the liquid, the Tait equation of state for water is used: $\rho _l(p)/(p+B)^{1/n_T} = const.$, here the Tait exponent is $n_{\mathrm T} = 7.15$ and the Tait pressure is $B = 305\,$MPa.

The simulations are carried out in full 3D, with the computational mesh of same size as the cuvette in the experiment. The originally coarse mesh with cells in Cartesian orientation is refined in the region of the circular disc shown in Fig. 1b. The disc is then cut out of the mesh, as detailed in Appendix C. The height of the disc is assumed as 1 mm. The coordinate origin is located in the top centre of the disc (matching the centre of the rectangular cuvette). The bubble site at $t=0$ is set to be at $(x=0,\,y=-r_b,\,z=d)$, as defined in Figs. 1a and 20. The mesh is refined in concentric spherical regions around the bubble site, starting from a distance of 3.5 mm for the first (outermost) refinement and ending at the 7th (innermost) refinement region with a distance of 30$\upmu$m to the bubble centre at $t=0$. The mesh is static and does not change over time for one simulation.

The minimum cell size is 3.1 $\upmu$m in edge length. The total number of cells is half a million with this approach. The resolution is sufficient to capture the bubble dynamics including the jetting. A convergence test showed that neither the bubble jetting dynamics nor the velocity of the jet experience significant changes when the cell size is decreased or the region of finer cells is increased in size. The peak pressure values of shocks, on the other hand, are likely underestimated with the present resolution. However, this is acceptable since the formation and propagation of shock waves are not intended to be extensively explored in this study.

The boundaries of the cuvette are set to be approximately non-reflecting (Poinsot and Lele 1992; OpenFOAM Wiki 2010). This is possible because the cell size at the cuvette walls is significantly larger than the size of a shock front. This results in the broadening of shock waves in space, potentially leading to erroneous results when they are reflected back to the bubble region. The solid boundary (i.e. the circular disc) has a vanishing normal gradient for the pressure and volume–fraction field, as well as a no-slip condition for the velocity.

In the experiments, a typical laser bubble might initially exhibit some asphericity due to the slightly elongated shape of the laser plasma. However, the bubble quickly loses most of this initial asymmetry during expansion, partly due to surface tension. The effect of the initial bubble shape on bubble jetting dynamics is negligible when compared to the influence of the nearby wall. Therefore, for simplicity, we initiated the laser-induced cavity as a spherical bubble seed with a radius of $R_{\mathrm{{init}}}=20\,{\upmu \text {m}}$ at the laser focus site. The internal pressure in the bubble is much higher than the pressure in the surrounding liquid, such that it expands rapidly. For the simulations in Figs. 4, 5 and 7, the parameters *d* and $r_b$ were read from the experiments; however, $r_b$ as well as the initial pressure of the bubble is adjusted so that there is a best match between the dynamics of the numerical bubble and the experimentally observed one. For a larger numerical parameter study over $r_b$ for a fixed $d=480\,{\upmu \text {m}}$ (compare Figs. 10, 11 and 12), the pressure in the bubble is set to 1.1 GPa, which has been chosen in such a way that the bubble would attain a maximum radius of $\sim 500$ $\upmu$m in unbounded liquid ($R_{\mathrm{{max,ub}}}$), corresponding to a volume $V_{\mathrm{{max,ub}}}=9.187\cdot 10^{-9}\,\textrm{m}^3$. The maximum bubble size, however, turns out to decrease slightly as the bubble seeding position approaches the solid boundary (see also, for example, Figure 2 in Ref. (Koch et al. 2023)).

The liquid is set at rest with an ambient pressure of 1 bar. The adaptive time step is limited by the flow Courant number with a maximum value of 0.2.

## Results and discussion

The results concerning the jetting dynamics of bubbles expanding and collapsing above a glass disc are presented through a combination of experimental x-ray holographic images, synchronous high-speed optical images, and renders from three-dimensional numerical simulations. Section 4.1 introduces to the peculiarities of bubble imaging by x-ray holography and to the image interpretation. Section 4.2 represents the core of this work which is focused on studying the effect of an off-centred bubble position on the jetting direction for bubbles produced at different stand-off distances $\gamma$. Subsequently, the effect of varying the distance of the bubble to the border of the disc is discussed in Sect. 4.3. Furthermore, some remarkable findings on internal gas/fluid structures, only visible under the x-ray illumination, are detailed in Sects. 4.4 and 4.5.

In the experiments, $R_D=4$ mm, and the bubbles were produced at radial distances around $r_b = 3$ mm, which results in $\alpha _r \sim 2$ for $R_{\mathrm{{max}}}\approx 500\,\upmu$m. It was not possible to explore larger variations of $r_b$ and $R_D$ due to technical issues (e.g. the existing restriction on the cuvette size imposed by the x-ray absorption in the liquid) and limitations in the available beamtime. However, a wider range of $r_b$ resp. $\alpha _r$ has been explored by numerical simulations, as given in Sect. 4.3.

The distance between the bubble seeding position and the cuvette walls in this experimental setup was always kept to $8\,R_{\mathrm{{max}}}$ or higher. The separation from the bubble to the stick which holds the rigid plate was even larger (see Fig. 1). Thus, the walls and the stick are not expected to play any significant influence on the bubble jetting dynamics, especially considering the close proximity of the surface of the plate. It is worth noting that the bubble seeding position might have slightly fluctuated for different values of $\gamma$. This is a consequence of using a relatively low numerical aperture in the beam focusing optics, as well as the partial blocking of the laser beam that occurs when the plate is placed very close to the laser focal spot. Accordingly, the exact position of the bubbles on the glass plate was obtained from the optical images in the direction of the beam and finely adjusted through the numerical simulations in the perpendicular direction.

### Interior of a jetting bubble

The gas–liquid interface of a jetting bubble can present quite complex shapes characterized by curved domes, rings and an internal liquid column. This makes the observation inside the bubble extremely difficult due to the refraction and reflection of light in the visible spectrum. For instance, when using traditional backlight illumination (shadowgraphy) the majority of the projected area of the bubble in the screen looks obscure in the high-speed recording (Koch et al. 2021). On the contrary, x-ray holography produces images provide a clearer view of the interior of the gas cavities and hence represent a great advantage relative to the traditional optical imaging. However, the x-ray holographic images cannot be interpreted exactly in the same way as conventional optical images. In the holograms, the phase shift induced by the liquid–gas structure of the sample on the transmitted x-rays results in distinctive features of the measured intensity pattern which are characteristic of near-field diffraction, which have to be understood before interpretation of the images.

To give a proper interpretation to the raw holograms, we compared them with simulated holograms derived from data obtained through computational fluid dynamics simulations together with an electromagnetic wave propagation model. Through these x-ray simulations, we gain insights into the origins of certain distinct and counter-intuitive characteristics found in the x-ray holograms. A more detailed explanation on this method of model-based fitting can be found in Appendix A.

**Fig. 3:**
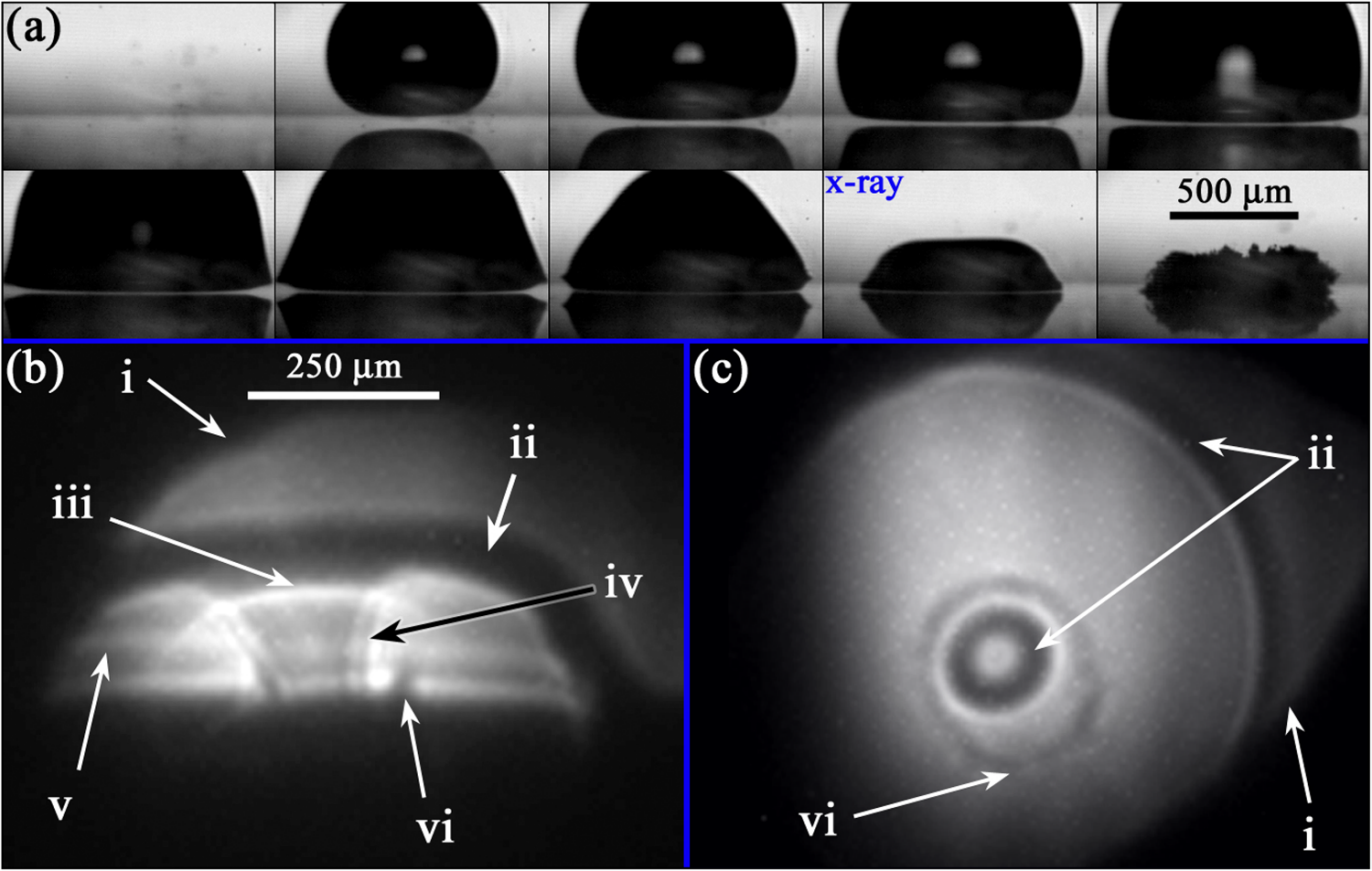
Optical and x-ray holographic imaging of a bubble jetting towards a rigid (glass) surface. **a** Optical image sequence of the bubble dynamics taken with 75 kfps (i.e. $13.3\,\upmu {s}$ between frames). Here, the stand-off distance is $\gamma =0.57\pm 0.03$. The lower panels present x-ray holographic images captured at the corresponding instant marked with the legend “x-ray” in (**a**). Panel **b** shows a side view of the jetting cavity as an X-ray hologram, while in (**c**) the jetting of a second bubble with similar dynamics is observed from a parallel view (normal to the glass disc). The x-ray light reveals the bubble interior, invisible in the optical shadowgraphs of (**a**). The arrows indicate some of the features commonly present in the holograms: i x-ray light blocked by the border of the hole of the laser mirror; ii thick interfacial line; iii bright arc; iv double shadow at the jet liquid column; v bubble pedestal stripes; and vi Blake splash

One example of the obtained x-ray images is presented in Fig. 3. Figure 3a shows a sequence of optical images on the jetting dynamics of a bubble produced at $\gamma =0.57\pm 0.03$, while some of the typical distinctive features observed in the corresponding x-ray holograms are enumerated in Fig. 3b and c. A characteristic that stands out in the x-ray images can be seen at the interface between the interior of the bubble and the water. There, a prominent contrast along the edges manifests as a wide outer dark border accompanied by a bright inner edge (ii). A similar contrast variation is also evident (although less pronounced) at the boundary between the gas phase and the inner jet (iv). These edge interferences (“edge enhancement”) are typical for the holographic recording. Another prominent feature found in the holograms is given by the downward-curving bright arc around the jet top (iii). This is a result of the torus-like shape assumed by the collapsing bubble on its upper portion, and it is well reproduced by simulations (see Appendix A).

In the perpendicular x-ray view of the bubble (i.e. Figure 3b), the visibility of the splash (vi) produced by the jet impact on the surface is hindered by a dark region in proximity to the surface. This is attributed to both the dark region of the bubble boundary and also to the strong contrast of the edge of the glass boundary which might overlap with certain physical features that occur in close proximity of the solid boundary (see Appendix A). As demonstrated in Fig. 3c, the splash is well discernible in the parallel x-ray view.

Figure 3b also displays the formation of “bubble pedestal stripes” near the surface (v). These stem from the kink in the outer bubble interface region that is projected through the bubble. This characteristic “bell shape” of the bubble forms during jetting for certain $\gamma$, due to a thin liquid boundary layer between bubble and surface (Lechner et al. 2020; Koch et al. 2021).

The x-ray illumination function is non-uniform, which leads to random background fluctuations in most images. Also the position of the illumination centre is subject to jitter. Both is due to the nature of the self-amplification of spontaneous emission (SASE) process, which underlies the XFEL radiation. For further discussion, see Appendix A or Ref. (Hagemann et al. 2021). In addition, as the X-ray beam passes the drilled mirror, the edges of the through hole cut into the x-ray beam in some images, see feature (i) in Fig. 3b, c. Due to slight angular adjustments of the mirror, we see an ellipsoid aperture.

### Jetting dynamics of cavities with non-axisymmetric boundary conditions at different stand-off distances

**Fig. 4:**
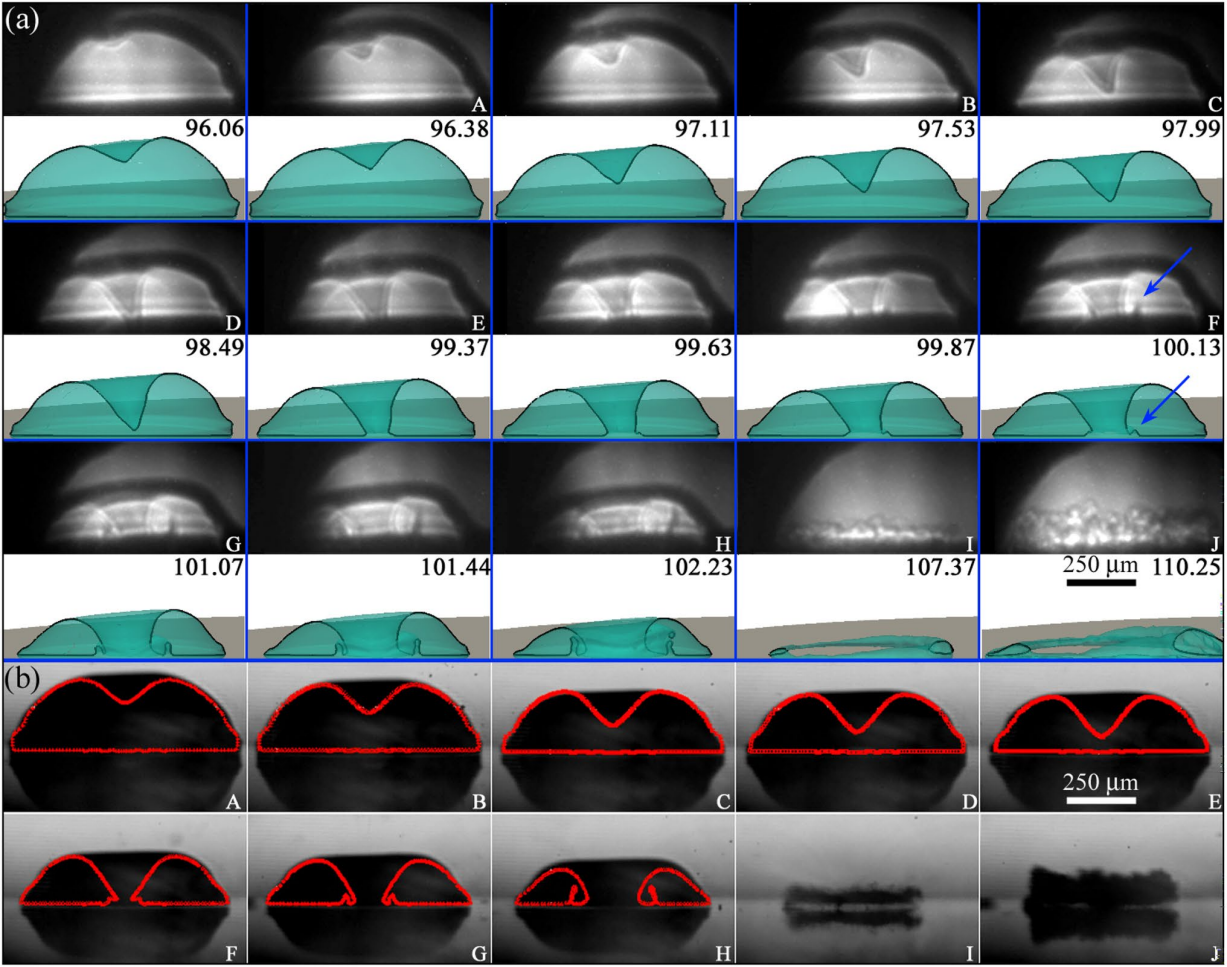
Bubble jetting observed from a perpendicular view, as explained in Fig. 1c, and contrasted with the full 3D numerical simulations, computed for a measured stand-off distance $\gamma =0.57\pm 0.03$. **a** x-ray holographic images of the jet dynamics and its impact on the solid surface. The images are compared with frames from the numerical simulations with time indicated in $\upmu {s}$ after the laser pulse. In those, the gas–liquid interface is highlighted by a black contour drawn along a plane crossing the centre of the bubble. The Blake splash can be clearly seen right after the jet impact, i.e. at $t\,\simeq \,100\,\upmu {s}$ (as pointed out by the blue arrows. **b** Optical images acquired simultaneously to the x-ray images from a direction orthogonal to the x-ray propagation. As the jetting points predominantly away from the camera, the bubble appears nearly symmetrical when viewed from this perspective. The two types of images are paired by the capital letters in the lower right corner of the frames. The red contours show the numerical simulations from the perspective of the optical camera, cut through the origin. Therefore, the moment of impact appears slightly retarded as compared to the according frames in (**a**). Multimedia view: (Video Fig. 3.avi)

The jetting dynamics of bubbles produced at different stand-off distances was studied through a series of individual x-ray holograms captured from multiple laser bubbles produced under the same conditions. The existing limitations on the equipment’s precision or the effective energy deposition of the laser on successive shots caused minor fluctuations on the bubble dynamics that translate into a small level of jitter in the temporal phase of the expansion/collapse cycle of the cavities. The holograms obtained from different single-pulse recordings were then arranged in a chronological order, creating the illusion of fluid motion by applying a technique known as “equivalent time sampling” (Philipp and Lauterborn 1998; Lindau and Lauterborn 2003; Koch et al. 2021). The process of sorting and assigning a specific time to each frame was performed by fitting the experimental bubbles to full 3D numerical simulations. This allows us to generate holography “movies” of the bubble jetting process with a variable equivalent frame rate ranging from one million frames per second (Mfps) to three Mfps, as shown in Fig. 4a. There, the irregular illumination pattern found in the different frames comes from a small level of flickering in the intensity and position of the x-ray beam.

Figure 4a presents a clear view of the oblique jet impacting the solid boundary (at $t\,\simeq \,99\,\upmu {s}$) and producing an asymmetrical Blake splash (Blake et al. 1998; Tong et al. 1999; Lechner et al. 2020; Bokman et al. 2023), invisible in the synchronous optical images shown in Fig. 4b. In the accompanying images from the simulation, the gas–liquid interface of the cavity is rendered in a semi-transparent turquoise colour, while the liquid is left white. The position of the jet tip and the evolution of the subsequent Blake splash are remarkably well reproduced by the numerical model. The general bubble dynamics and the formation of an oblique jet resembles the numerical results (obtained with the BIM) reported by Wang et al. (2020) for a case where a bubble collapses near a corner defined by two rigid walls. It is worth noting that in Fig. 4b, the red contours are cut through the centre of the initial bubble, specifically in a direction perpendicular to the x-ray image plane. Consequently, due to the oblique nature of the jets, there appears to be a delay in the jet dynamics displayed in panel (b) when compared with the corresponding frames in panel (a) of Fig. 4.

The numerical fitting of the x-ray holograms with the CFD model brings the possibility of performing a realistic and precise assessment of the jet’s speed, which is believed to be closely related to the extent of potential damage produced at the point of impact. This technique is particularly useful for interpolating the dynamics of experimental bubbles when the phenomena being studied occur at higher speeds than the sampling rate utilized in the holograms, e.g. to characterize fast jets or the bubble collapse. In the example of Fig. 4, the simulations indicate that the jet reaches a rather moderate speed of $72\,$m/s right before the impact.

After the jetting, the toroidal bubble collapses asymmetrically, i.e. starting from the side closer to the boundary and then progressing towards the centre of the disc. As demonstrated in panels “I” and “J” of Fig. 4, the cavity experiences significant fragmentation during its implosion towards the surface, subsequently expanding as a bubbly ring with a complex gas–liquid structure.

**Fig. 5:**
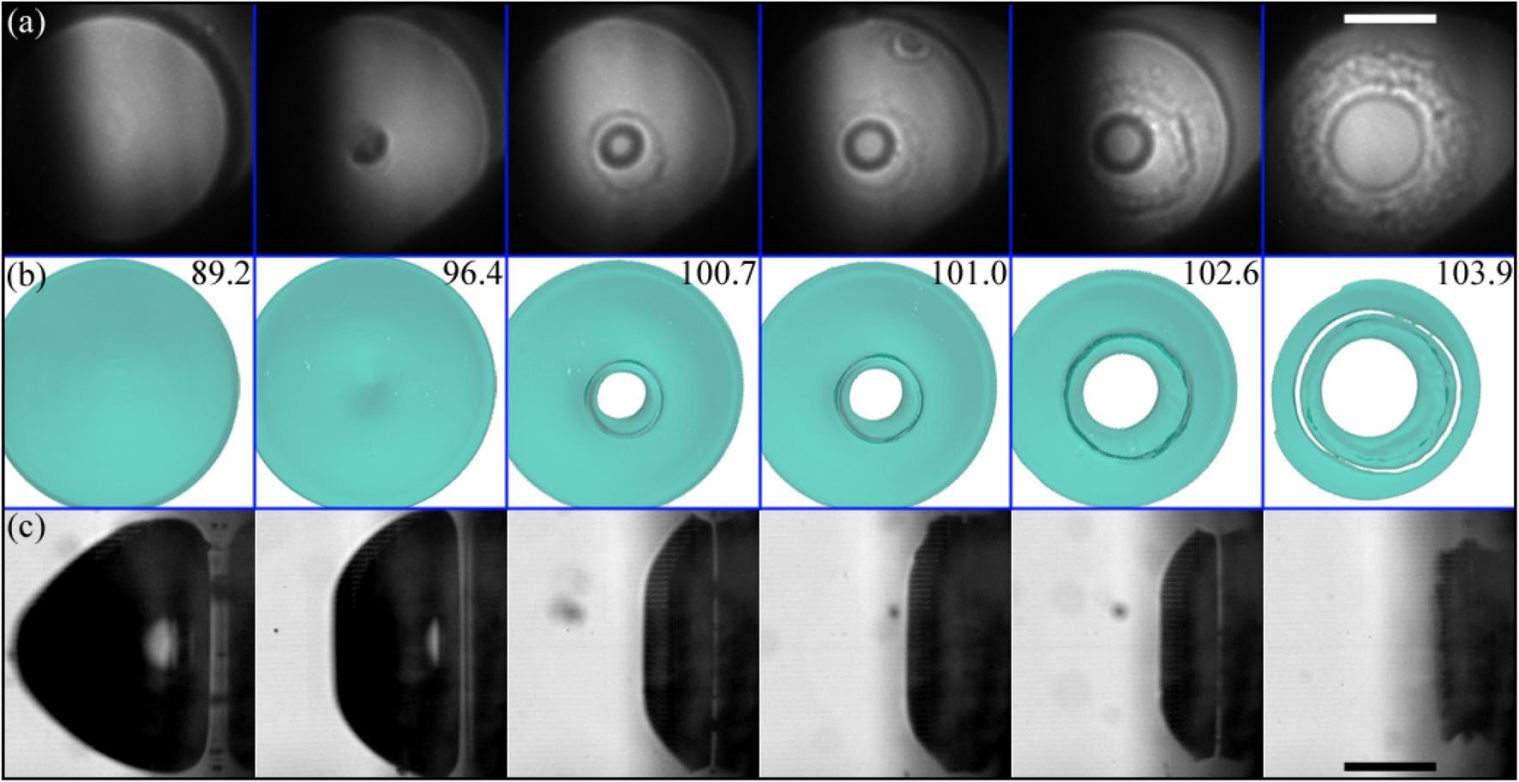
Parallel view of the bubble collapse as the jet hits the glass plate for a case with $\gamma =0.56\pm 0.03$. The scale bars represent $250\,{\upmu \text {m}}$. **a** X-ray images of the jet impact observed from a parallel view (see Fig. 1c). The asymmetry of the splash and the cavity collapse is clearly visible from this perspective. For example, the splash is predominantly directed towards the lower right corner of the panels in (**a**) and (**b**). Here the time in $\upmu {s}$ was obtained by matching the experimental frames with the simulations presented in panel (**b**). The gas–liquid interface in the simulation is rendered in a semi-transparent turquoise colour, while the liquid is left white. **c** Synchronous optical images taken perpendicular to the glass disc and orthogonal to the x-ray beam direction

Further details on the dynamics of the splashing and the later rupture of the toroidal bubble can be examined in the parallel view of the phenomenon presented in Fig. 5. The view from this perspective makes evident that the jet can be detected even on the early stages of its formation as a shaded region on the holograms. This perspective also highlights the asymmetry of the splash and provides an account of the height of its crown through the thickness and darkness of the splash contour line, which becomes more pronounced where the crown reaches greater heights.

**Fig. 6:**
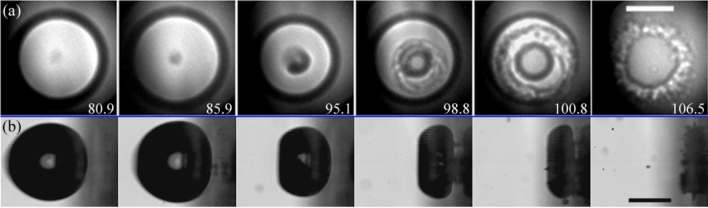
**a** X-ray holographic images in parallel view of the jet impacting the rigid boundary at $\gamma =0.84\pm 0.03$. Here the bubble is located around $r_b=2.8$ mm from the centre of the plate (i.e. $\alpha _r=2.4$). The scale bars represent $250\,{\upmu \text {m}}$, the numbers indicate time in $\upmu {s}$. **b** Optical images in side view corresponding to the frames in (**a**), taken perpendicular to the glass disc and orthogonal to the x-ray beam direction

Figure 6a exhibits a similar measurement taken for a slightly higher stand-off distance (i.e. $\gamma =0.84$). In this case, the bubble is not in contact with the surface at the instant of jet impact. As the jet progresses further towards the disc, the asymmetry of the Blake splash becomes even more evident. For instance, this is seen in the non-central location where the jet touches the surface at $t=98.8\,\upmu {s}$ in Fig. 6b.

**Fig. 7:**
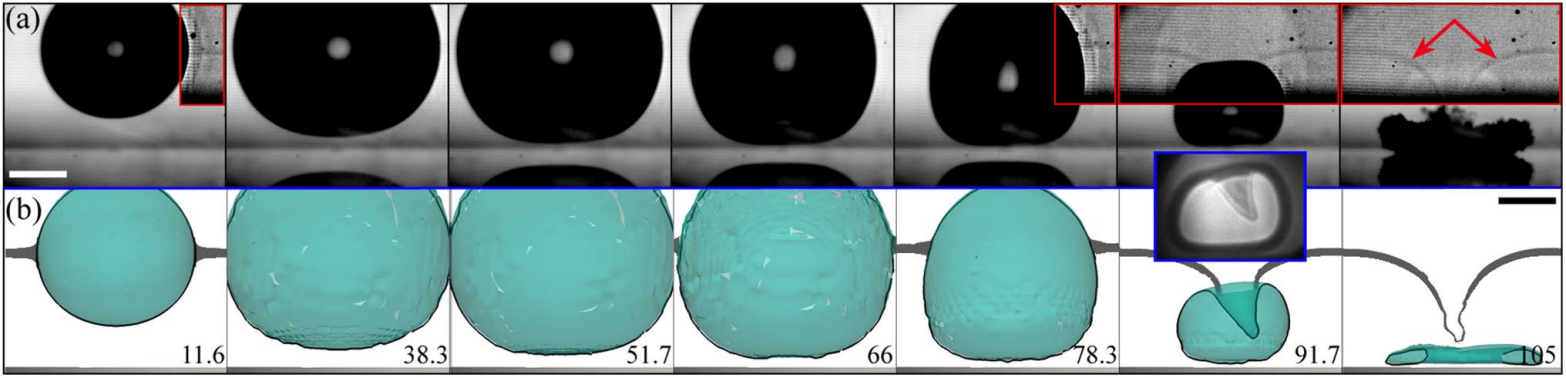
Perpendicular view of the jetting of a bubble with a stand-off distance of $\gamma =0.99\pm 0.03$. The optical images presented in (**a**) are compared with full 3D simulations in (**b**). The inset framed in blue presents the x-ray image corresponding the optical frame above to show the excellent agreement between experimental and numerical results. In the insets framed in red, the contrast of a section of the optical images was increased to highlight the generation of micrometric bubbles arranged in a line along the centre of the focused laser beam. The displacement of these tiny bubbles reveals the liquid flow induced by the jet (see red arrows). The shift of this line of small bubbles is reproduced by a synthetic dye advection visualization in the numerical simulations (grey lines above collapsed bubble in the last frame, bottom row). The numbers represent time in $\upmu {s}$ and the scale bars are $250\,\upmu {m}$ long

The investigation of jet tilting for a value of $\gamma$ close to unity was further explored through comprehensive 3D numerical simulations, as depicted in Fig. 7. Interestingly, we noticed that the passage of the laser pulse results in a faintly darker line in many of the optical images. This phenomenon is produced by the appearance of a linear trail of micrometric or sub-micrometric bubbles along the centre of the laser beam cone of light (Rosselló and Ohl 2021). The regions in the images with increased contrast (outlined in red) in Fig. 7a show how these tiny bubbles are dragged by the flow induced by the laser cavity collapse, serving as tracers of the liquid displacement, as corroborated using a dye advection visualization (Jobard et al. 2002; Laramee et al. 2004; Koch et al. 2023) in the numerical simulations in Fig. 7b. In this figure, the grey horizontal layer in the first frame represents the secondary laser-induced bubbles advected with the flow, assuming the shape of the grey warped layer in the last two frames. The corrugations observed in the bubble interface in the simulation frames at maximum extension result from the interpolation of an oblique cut across computational cells and should not be mistaken as artefacts.

**Fig. 8:**
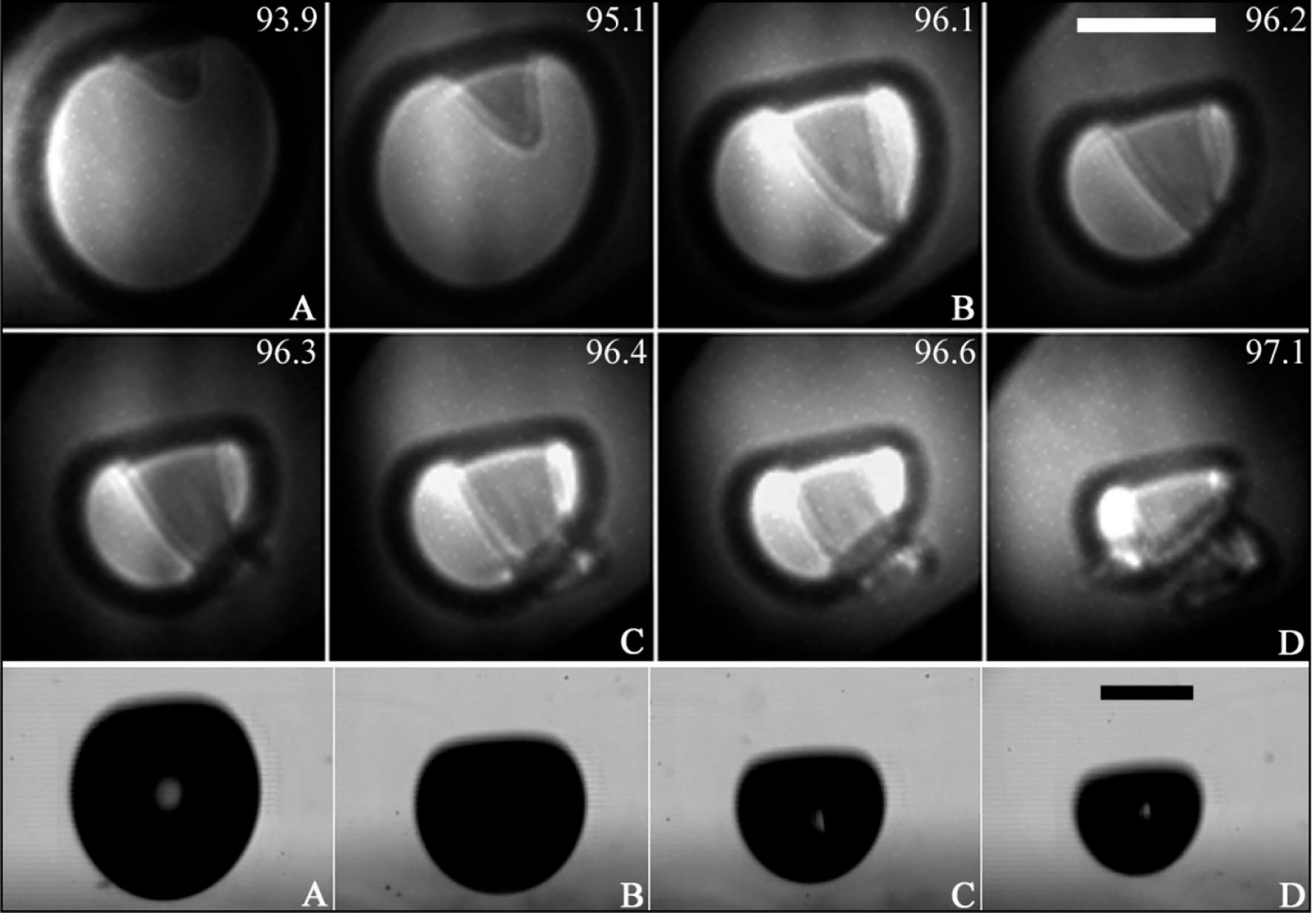
X-ray holographic images for a bubble with a stand-off parameter $\gamma =1.28\pm 0.03$ located at a radial distance $\alpha _r = 2.0 \pm 0.4$ from the border of the glass disc. The shadowgraph images in the bottom row are $90^{\circ }$ tilted and the jet is pointing to the camera. Corresponding frames are marked with the same capital letters. Here, the optical frames are advanced 690 ns with respect to the x-ray holograms. The scale bars represent $250\,{\upmu \text {m}}$ and the numbers time in $\upmu {s}$. Multimedia view: (Video Fig. 7.avi)

As the distance between the bubble seeding point and the plate is further increased, the effect of the anisotropic boundary conditions on the jetting dynamics becomes increasingly noticeable. For instance, if the stand-off parameter used in Fig. 4 is doubled while maintaining $\alpha _r$ fixed, we obtain a cavity that remains spherical during the expansion phase to later produce an oblique jet which forms an angle (with respect to the vertical direction) as shown in Fig. 8.

The cases with values of $\gamma$ ranging from 1.3 to 2 are distinguished by a relatively wider jet (i.e. with respect to the bubble size) that penetrates the bubble without being hindered by the rigid boundary. In the current anisotropic scenario, this leads to the formation of a non-axisymmetric toroidal cavity with variable cross section.

**Fig. 9:**
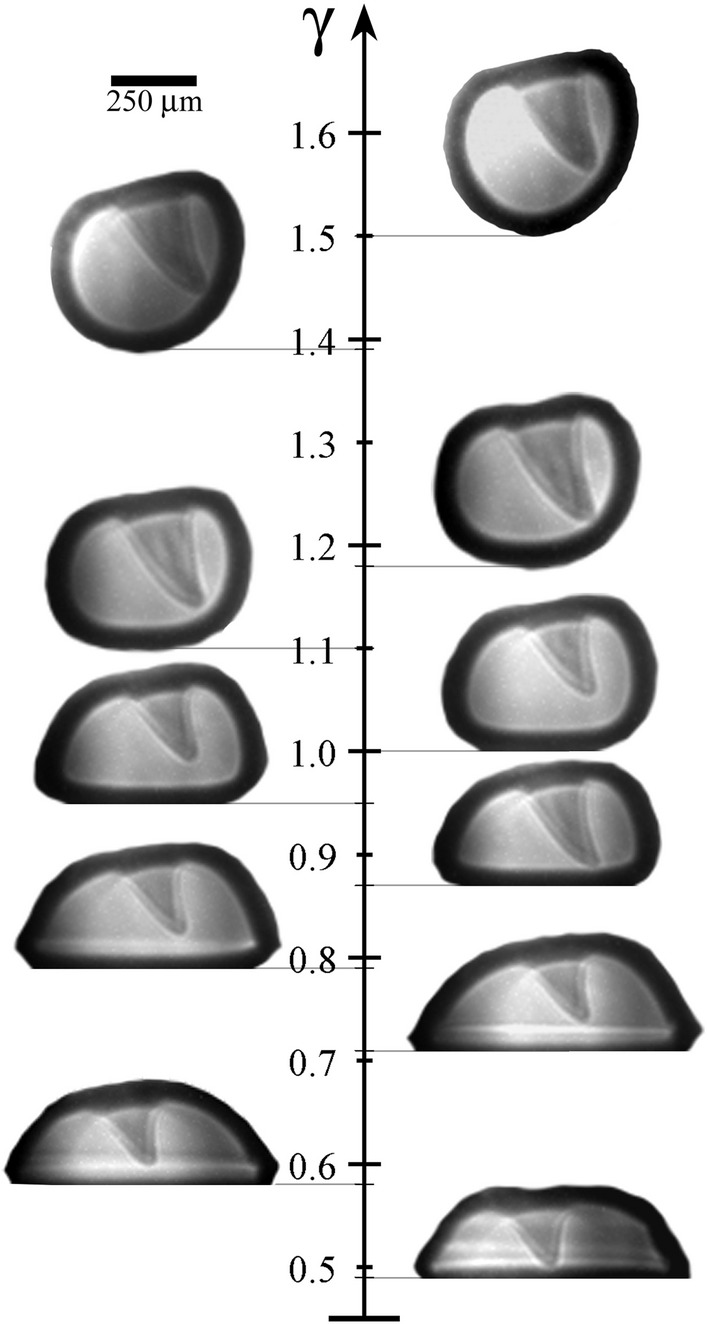
Oblique jetting of bubbles produced at different stand-off distances for a fixed radial position $\alpha _r=2$. The parameter plot makes evident the changes occurring on the bubble shape and the jetting direction when $\gamma$ is varied. Here, the jet evolution is captured instants before the jet pierces the lower wall of the cavity. As the bubble is generated closer to the rigid boundary the orientation of the jet becomes progressively aligned with the surface’s normal direction

The changes observed on the oblique jet/bubble dynamics for the range of $\gamma$ values previously discussed are summarized in Fig. 9. The parametric plot illustrates the evolution of the jetting bubble shape with increasing stand-off distance, showing the significant impact of $\gamma$ in the bubble morphology, for instance producing a progressive flattening of the gas cavity. However, the rather minor changes observed at higher values of $\gamma$ suggest that its influence becomes gradually weaker as the stand-off distance is further increased. In this regard, the angle formed by the jet tip with the vertical direction becomes slightly larger when the bubble is placed away from the surface, i.e. the oblique jet gradually deviates from the vertical direction. This suggests the existence of a nonlinear relationship existing between the stand-off distance $\gamma$ and the influence of the rigid boundary on jet development. This implies that when the bubble is in close proximity to the surface, even minor variations in $\gamma$ exert a substantial impact on the bubble dynamics, in contrast to similar changes when the bubble is far from the boundary. This observation was confirmed through numerical simulations.

### Effect of the radial position $r_b$ on the bubble dynamics analysed through numerical simulations

In the preceding section, we explored the effect of $\gamma$ on the bubble/jetting dynamics. Let us now focus on examining the influence of the seeding radial position, denoted as $r_b$. To quantify the impact of this parameter on the jetting, and in absence of sufficient experimental data, we performed systematic full 3D numerical simulations shifting $r_b$ from the axisymmetric case (i.e. $r_b=0$) to the border of the glass rigid plate at $r_b=4\,$mm. Here the value of the dimensionless stand-off is fixed to $\gamma =0.99$, which corresponds to the experimental case presented in Fig. 7.

**Fig. 10:**
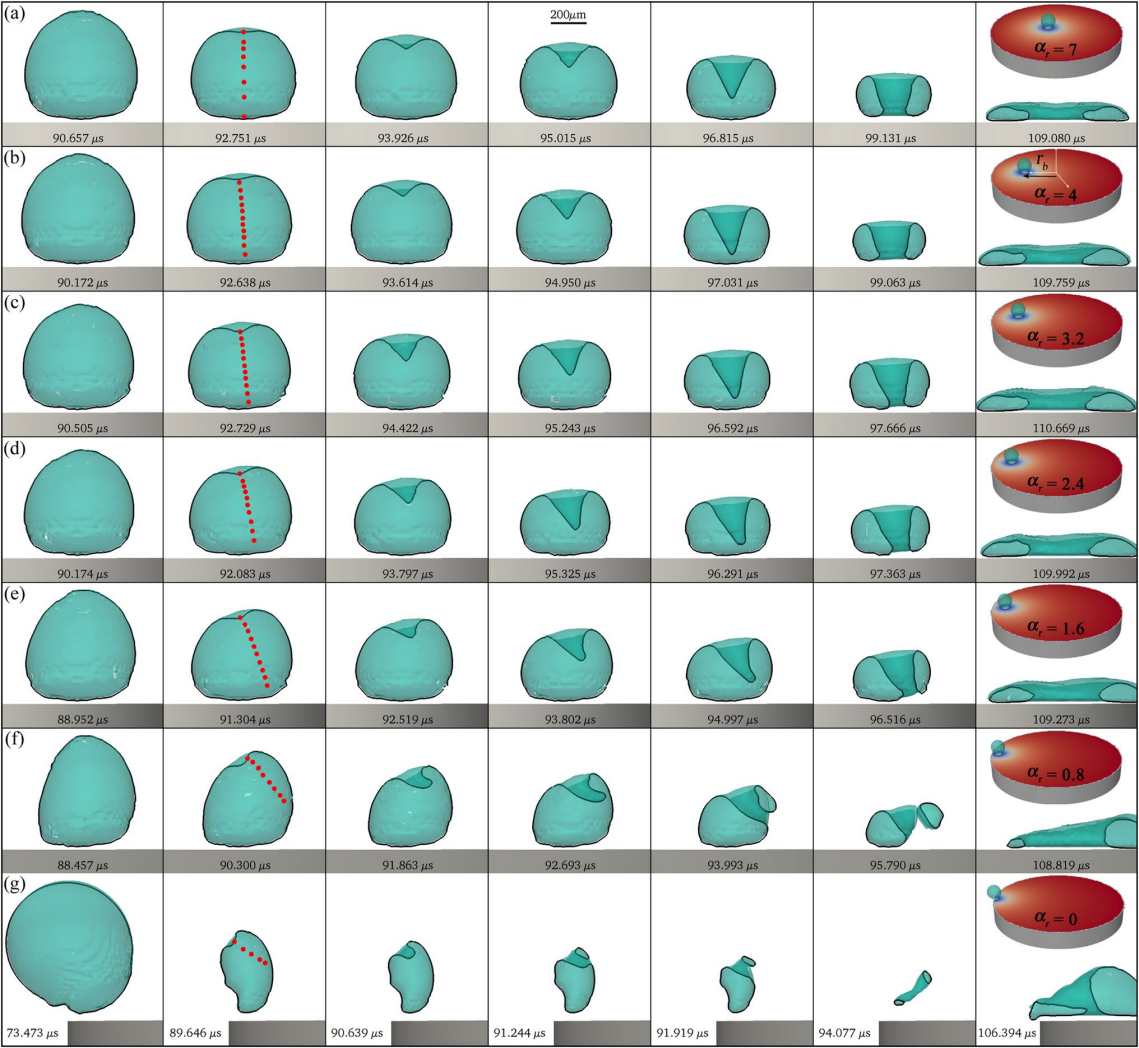
Three-dimensional numerical simulations of the bubble jetting at different positions on the disc. The parameters for the simulation were taken from the experimental case presented in Fig. 7 (i.e. with $\gamma =0.99$). The initial radial position of the bubble was set at **a** $\alpha _r=7$ ($r_b=0.5$ mm), **b** $\alpha _r=4$ ($r_b=2.0$ mm), **c** $\alpha _r=3.2$ ($r_b=2.4$ mm), **d** $\alpha _r=2.4$ ($r_b=2.8$ mm), **e** $\alpha _r=1.6$ ($r_b=3.2$ mm), **f** $\alpha _r=0.8$ ($r_b=3.6$ mm), and **g** $\alpha _r=0$ ($r_b=4.0$ mm). The red dots in the second column track the position of the jet tip from its formation until it pierces the cavity. The inset in the last column shows the full scenario, indicating the bubble position and with colour-coded pressure on the disc surface

The results are summarized in Fig. 10. There, it is possible to observe that the axisymmetric case ($\alpha _r=8$) does not deform considerably, however deviations from axial symmetry become noticeable down to $\alpha _r\approx 4$. The experimental case in Fig. 7 corresponds to $\alpha _r=2$, i.e. between rows (c) and (d). The simulations demonstrate the growing impact of the proximity of the disc boundary on some features of the jet temporal evolution, particularly its direction. As a general rule, the jet is bent inwards, i.e. towards the centre of the disc. The downward infiltration of liquid into the cavity originates from an involution of the bubble wall at its cusp, at approximately $t=93\,\upmu$s for the cases with higher $\alpha _r$ and slightly earlier as the bubble approaches the border. As the value of $\alpha _r$ decreases, the influence of the disc limits becomes more pronounced, leading to the creation of a curved filament. Remarkably, even though the jet tip is following a straight line, as depicted by the red dots in the second column of Fig. 10, the whole jet structure exhibits a curved shape.

**Fig. 11:**
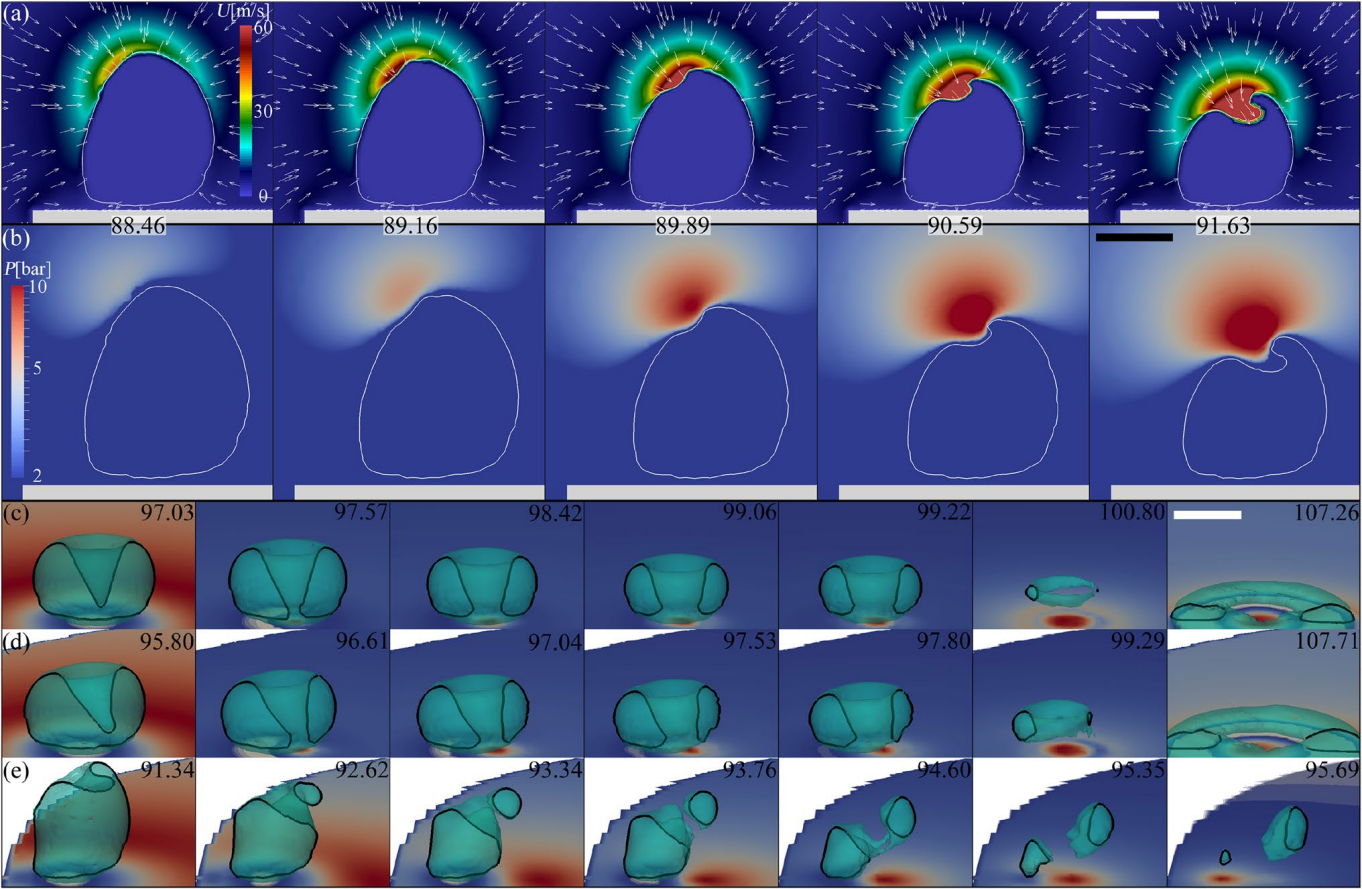
Numerical simulations of the velocity field (**a**) and the pressure distribution (**b**) around the bubble during the formation of the oblique jet for a case with $\alpha _r=0.8$ ($r_b=3.6$ mm) and $\gamma =0.99$. Panels **c**, **d** and **e** present the temporal evolution pressure produced at the solid boundary by jetting bubbles at different positions on the disc. **c** $\alpha _r=4.0$ ($r_b=2.0$ mm), **d** $\alpha _r=2.0$ ($r_b=3.0$ mm) and **e** $\alpha _r=0.4$ ($r_b=3.8$ mm). The numbers indicate time in $\upmu {s}$. The colours represent pressure on the disc, ranging from 0 Pa (blue) to a maximum pressure that changes for each time step. In each panel, the scale bars represent $250\,\upmu {m}$

Essentially, the formation of an oblique jet originates from the uneven inflow during collapse, as illustrated in panel (a) of Fig. 11. There, we can see that the liquid can flow almost unhindered from the cavity side closer to the disc border, resulting in a flattening on that side of the bubble which becomes increasingly pronounced as the bubble collapse accelerates. At the same time, the pressure in the liquid surrounding the bubble wall is higher on one side of the bubble cusp, producing in a localized depression that leads to the intrusion of liquid into the bubble interior, i.e. forming the oblique jet (see Fig. 11b).

The lack of axial symmetry of the jet dynamics has also consequences on the collapse of the toroidal gas cavity. For instance, panels (c), (d) and (e) of Fig. 11 show how the gas phase collapses into the solid surface unevenly, i.e. starting from the side which is closer to the border of the disc, meaning that certain parts of the bubble collapse later than others. Furthermore, it can occur that different portions of the cavity begin to re-expand before others reach their maximum compression. An asymmetrical collapse also results in non-uniform acoustic emissions, which might lead to acoustic pressure focusing in regions other than the centre spot below the bubble, potentially causing surface damages (Philipp and Lauterborn 1998; Gutiérrez-Hernández et al. 2022; Reuter et al. 2022; Dular and Ohl 2023). In the axisymmetric case, the downwards jet generates a stagnation point right below the position where the bubble was seeded by the laser-induced plasma. As a consequence, an over pressure builds up on the surface that can easily reach a few MPa of peak value (Lechner et al. 2019). This elevated pressure persists throughout the duration of the jet, which typically lasts for approximately $5\,\upmu s$ in the case of bubbles with a maximum radius of around $500\,\upmu m$. In this regard, the simulations reveal an interesting observation: the temporal evolution of the over pressure produced at the stagnation point after the jet impact is very similar for all the cases; however, in the case of an oblique jet, the location of maximum pressure is not fixed throughout the jetting process, as shown in panels (c), (d) and (e) of Fig. 11. As the parameter $\alpha _r$ is reduced, the increased tilting of the jet results in an increased motion of the stagnation point, i.e. the spot of maximum pressure at the boundary. Caused by the inward bending of the jet, the stagnation point starts nearer to the plate’s centre, but then shifts towards the bubble’s initial distance $r_b$ during the ongoing collapse.

**Fig. 12:**
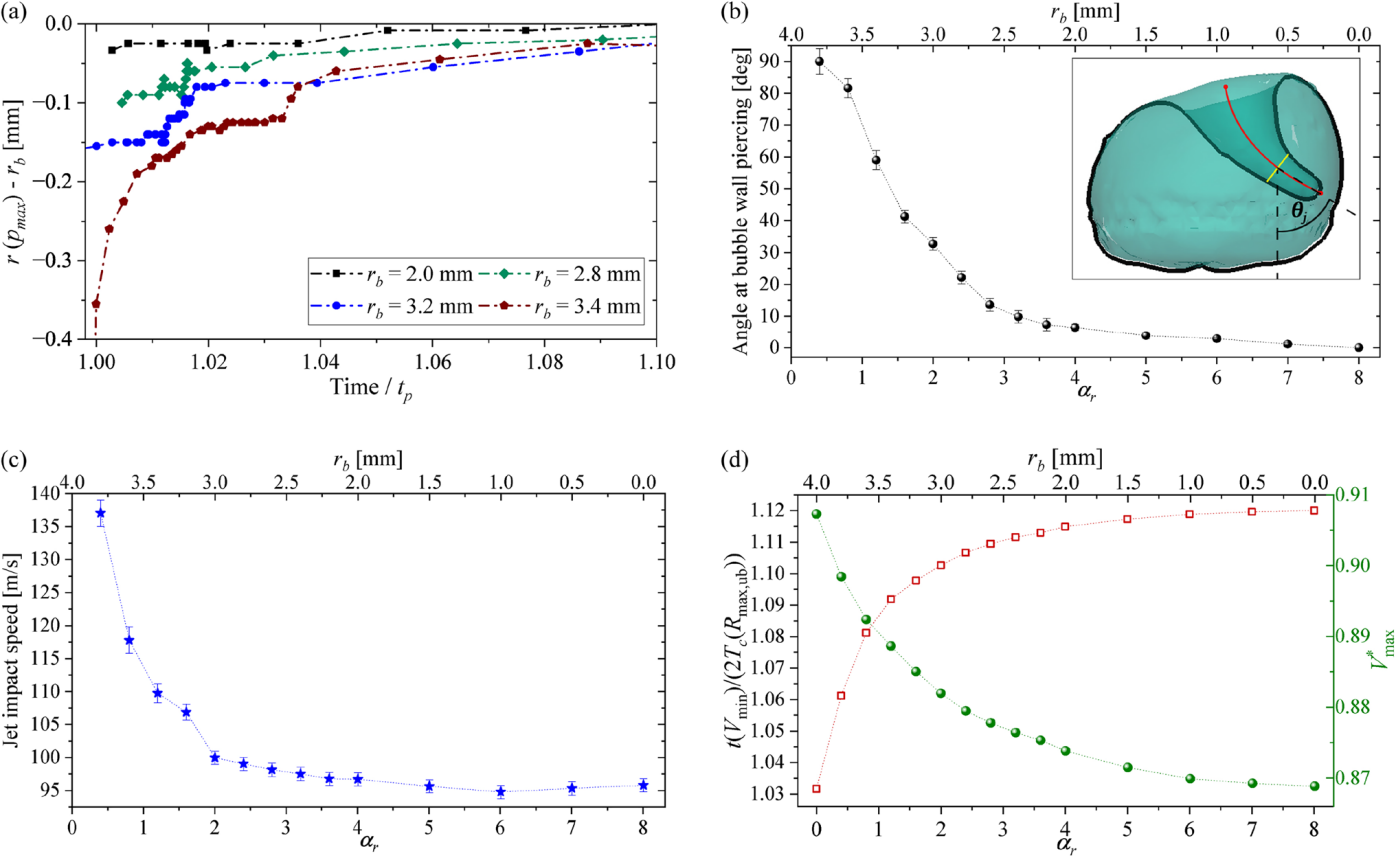
Bubble jetting parameters obtained from simulations with different $\alpha _r$ and a fixed $\gamma =0.99$, as shown in Fig. 10. **a** Radial location of the maximum pressure spot in the disc’s surface as a function of the time, which is normalized with the instant when the jet pierces the lower side of the gas cavity in each case (i.e. $t_p$). **b** Jet direction, as illustrated in the inset. **c** Jet speed at bubble piercing measured at the jet tip. **d** Prolongation factor in the collapse time of the cavities (red hollow markers) and normalized volume (green markers)

The movement of the stagnation point for bubbles originated at different values of $\alpha _r$ is depicted in Fig. 12a. It is worth clarifying that even when the cell size in the 3D simulations is not refined enough to accurately estimate the peak pressures on the disc surface, the observed change in its location is not influenced by minor variations in the mesh size. In all the cases, a moving maximum pressure might have consequences on the erosion scenario acting on the solid surface. For instance, the local duration of the peak pressure would be reduced, leading to the expectation of less material damage. However, a spatial shift of the peak pressure position might induce an additional shear force and thus cause additional lateral material loads.

The changes in the tilting of the jet when varying the radial position of bubble seeding have been extracted from the 3D numerical simulations, as detailed in Fig. 12b. In that analysis, the angle between the jet tip and the vertical direction ($\theta _j$) was measured at the moment just before the jet pierces the inner cavity wall. This measurement was performed by drawing a line connecting the tip and the midpoint of the jet width at the final third of its overall length (marked with a red line in the inset of Fig. 12b). The influence of the plate boundary on the jetting direction exhibits a nonlinear relationship with the parameter $\alpha _r$. Specifically, the numerical results reveal that the tilting effect is relatively minor for the central regions of the disc (i.e. large $\alpha _r$), however, it becomes increasingly significant when $\alpha _r$ decreases below about 3. As previously discussed, the asymmetric boundary exerts a significant influence, not only on the jet but also on the overall dynamics of the cavity. One specific example of this is the acceleration of the jetting and collapse as the bubble approaches the border of the plate. The speed of the jet, measured just before touching the bubble wall, demonstrates a similar dependence on $\alpha _r$ as the angle at which the jet pierces the bubble wall. Figure 12c shows that tilted jets exhibit a higher impact speed compared to cases where the jet is directed straight at the surface.

The acceleration of the collapse was measured using the prolongation factor, defined as $t(V_{\textrm{min}})/(2, T_c(R _{\textrm{max,ub}}))$, where $t(V_{\textrm{min}})$ is the collapse time of the cavity and $T_c(R_{\textrm{max,ub}})$ is the Rayleigh collapse time (Plesset and Chapman 1971). Figure 12d depicts a reduction in this dimensionless time as the bubble approaches the edge of the disc. The change in the prolongation factor can be attributed to variations in flow restriction around the bubble at different values of $\alpha _r$.

The normalized maximum volume ($V^*_{\mathrm{{max}}}$) of the bubbles near the border of the disc, on the other hand, is higher than in the middle of the disc, as detailed in Fig. 12d (i.e. the green axis). In principle, this result might appear contradictory because, according to the Rayleigh collapse time (defined in Sect. 3.1), larger bubbles are expected to take more time to collapse. From the simulations, it is observed that the bubbles in the centre region of the disc are hindered to reach the full volume of the unbounded case due to the boundary restriction. At the border of the disc the restriction is reduced, so the bubble size approaches the one of the unbounded case.

**Fig. 13:**
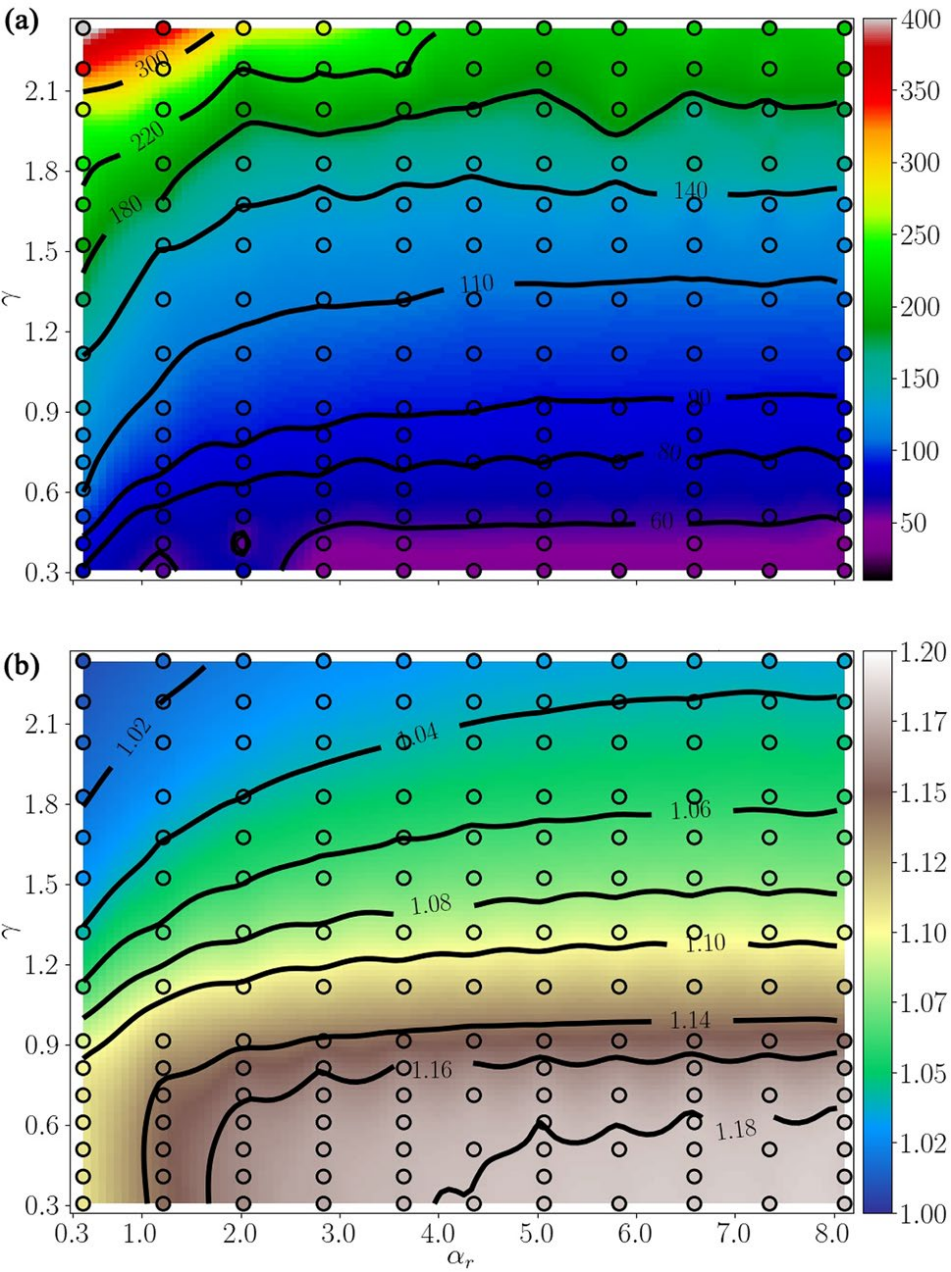
Simulated parameter maps are presented for various $\alpha _r$ and $\gamma$ values. These maps depict a more comprehensive representation of **a** the jet speed at bubble piercing, measured in m/s, and **b** the prolongation factor in the collapse time of the cavities. The contour plots were generated from the simulations denoted by the circular markers

A deeper understanding of the combined and individual influence of the stand-off distances on the jetting dynamics can be attained by examining parameter maps, such as those shown in Fig. 13. The figure shows maps of the jet speed and the prolongation factor in the collapse time of bubbles, obtained by varying the values of $\alpha _r$ and $\gamma$ in the numerical simulations across a range that encompasses the parameter values utilized in the experiments. From Fig. 13a, it becomes evident that the jet velocity achieves higher values for bubbles located at a radial position near the border of the disc (i.e. $\alpha _r=0$), as suggested in Fig. 12c for the case with $\gamma =0.99$. At the same time, the speed of the oblique jets also increases with the vertical distance to the plate (i.e. $\gamma$), similar to what is observed for the collapse of axisymmetric bubbles (Lechner et al. 2020).

Figure 13b displays the map of the prolongation factor in the collapse time computed as $t(V_{\textrm{min}})/(2\, T_c(R _{\textrm{max,ub}}))$. In line with the observed trend in the jet speed, the bubble dynamics are slower in regions near the centre of the disc. In contrast, bubbles placed over its periphery or away from the solid surface (i.e. for higher stand-off distances $\gamma$) exhibit a prolongation factor close to unity, corresponding to the unbounded case of a Rayleigh bubble.

The maps for both the jet speed and the prolongation factor confirm that, in general, the effect of the disc border proximity becomes increasingly relevant for $\alpha _r \lesssim 3$. Beyond $\alpha _r \gtrsim 5$, the scenario approaches similarity to the case of a bubble in a semi-unbounded liquid. The values of the jet speed for $\alpha _r \approx 8$ are in good agreement with those reported by Lechner et al. (2020) for an axisymmetric case, i.e. a bubble collapsing near an infinite solid planar boundary.

### Internal structure of a jetting bubble at $\gamma \gtrsim 2$

**Fig. 14:**
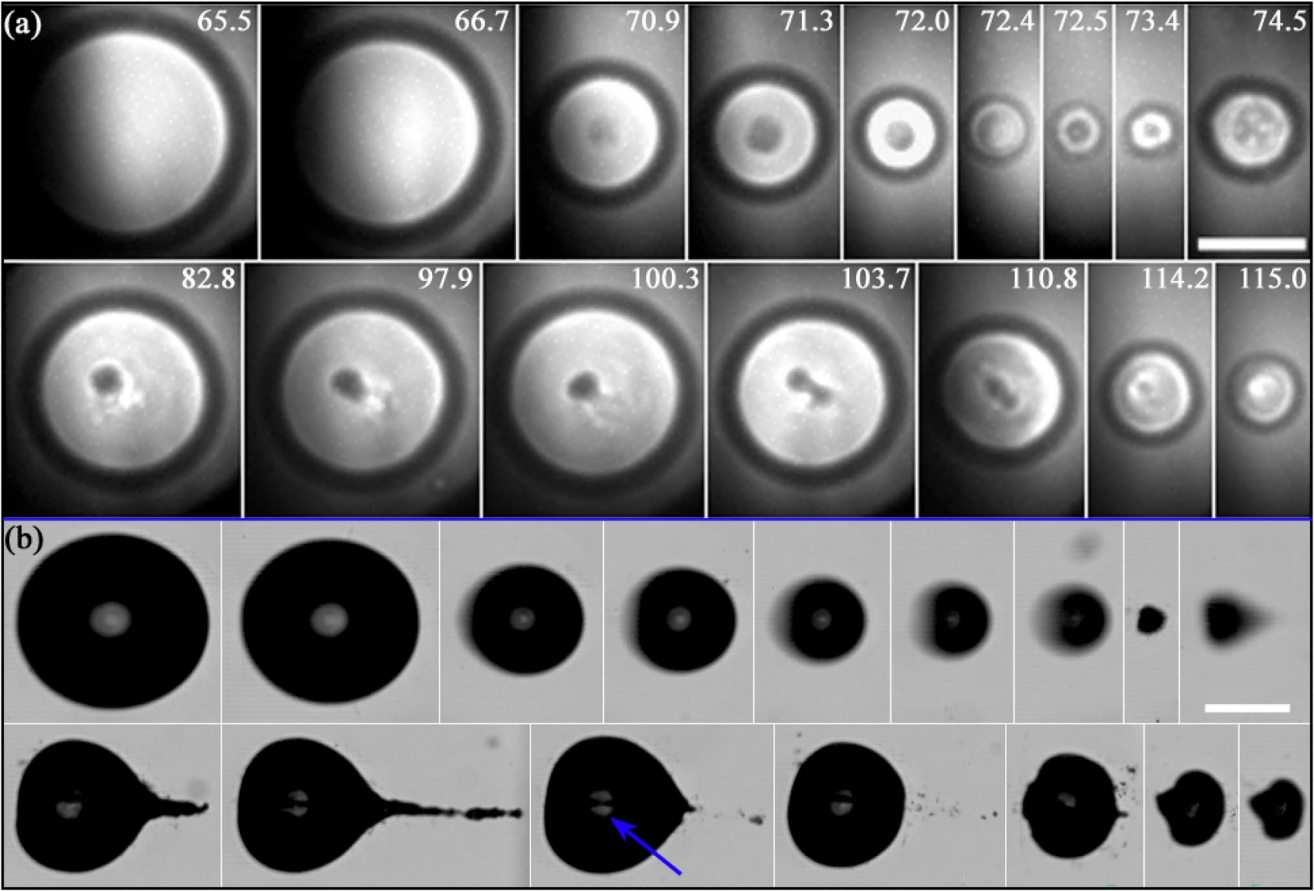
Bubble jetting at a stand-off distance of $\gamma \simeq 2.5$. **a** X-ray images acquired setting the glass disc normally to the beam direction as detailed in Fig. 1c, i.e. in the parallel view configuration. **b** Optical images taken from the side, perpendicular to the x-ray direction. The blue arrow highlights the partial visibility of the thin jet inside the gas cavity. The scale bars represent $250\,{\upmu \text {m}}$. The numbers indicate time in $\upmu {s}$

**Fig. 15:**
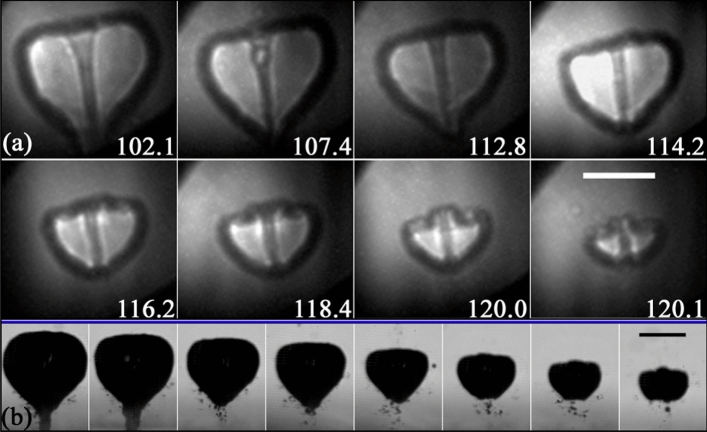
Second collapse of a cavity after the development of a “weak” jet at $\gamma =1.98\pm 0.03$. For values of $\gamma \gtrsim 2$ the jetting results in a thin liquid filament formed during the gas re-expansion phase. The particular shape acquired by the gas cavity during this second oscillation cycle is indistinguishable from the shadowgraphs alone. The scale bars represent $250\,\upmu {m}$. The numbers indicate time in $\upmu {s}$

When the stand-off distance is $\gamma \gtrsim 2$, the influence of the boundary on the bubble dynamics gradually diminishes, leading to a “weak” jetting regime characterized by the growth of a thin and relatively long liquid filament inside the cavity during its rebound phase (Lauterborn and Bolle 1975; Rosselló et al. 2023). This experimental scenario is depicted in Figs. 14 and 15 from two orthogonal perspectives. Figure 14a presents a sequence of x-ray holographic images showing the collapse and rebound of a bubble for $\gamma \simeq 2.5$ taken from a perspective parallel to the jetting direction. These x-ray images are complemented by optical images taken from the side view (see Fig. 14b) meant to provide a comprehensive interpretation of the holograms.

Within this range of stand-off distances, the bubbles adopt a concave form during the collapse process (e.g. the meniscus formed at $t=74\,\upmu {s}$ in Fig. 17). This distinctive characteristic is evident in the first row of panel (a) of Fig. 14, as observed by the ring-shaped appearance acquired by the bubble in the final stages of collapse. Following the rebound, the optical images clearly show the development of a liquid filament that penetrates the bubble and extends well beyond the size of the gas cavity. This elongated fluid structure is visible as a darker line segment in the holograms.

The second collapse of the cavity in the weak jet scenario also displays an interesting structure formed when the gas phase shrinks around the liquid filament, as shown in Fig. 15. The intricate flow dynamics leading to this particular shape is discussed in detail in Ref. (Rosselló et al. 2023).

### Formation of cavitation bubbles during the jetting

**Fig. 16:**
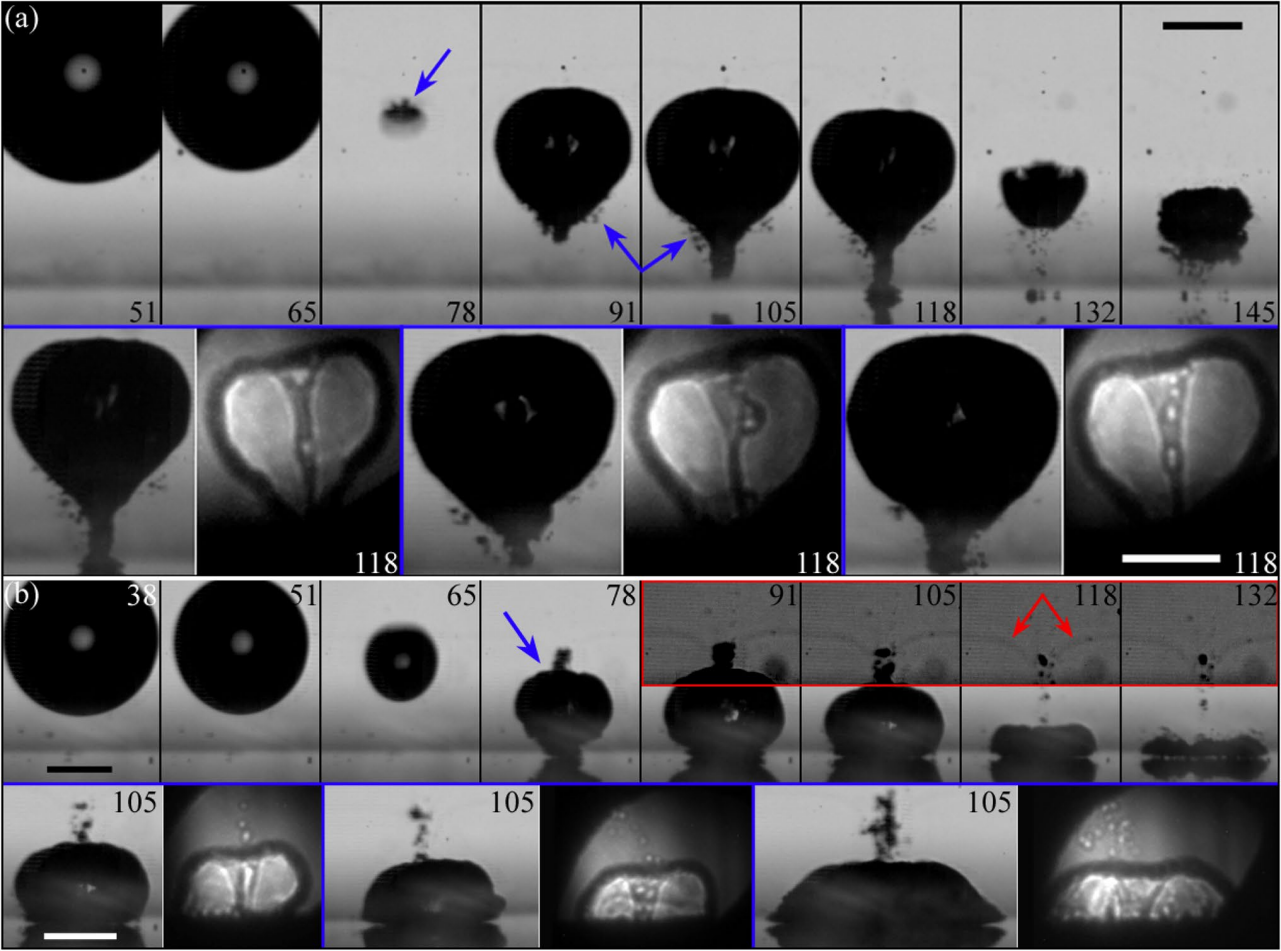
Surface instability of cavitation bubbles and its effect on the jetting. In both panels (**a**) and (**b**) the top row presents high-speed videos of a jetting cavity which evidences the bubble fragmentation at its collapse, here indicated by the blue arrows. The lower row compares optical (left) and x-ray (right) images taken simultaneously. The x-ray images reveal internal gaseous structures inside the jetting bubbles. Here, the stand-offs were $\gamma =1.87\pm 0.05$ for cases in (**a**) and $\gamma =1.36\pm 0.05$ in (**b**). In this last panel, the region framed in red highlights the displacement of tiny secondary laser-induced bubbles (initially arranged as a horizontal line). The scale bars represent $250\,{\upmu \text {m}}$ and the numbers indicate time in $\upmu {s}$

For some values of $\gamma$, the violence of the bubble collapse is such that the cavity undergoes a severe fragmentation promoted by the Rayleigh–Taylor instability and the strong acoustic emissions launched at the cavity collapse. An intriguing observation, which can only be discerned from the x-ray images, is that the flow generated during the jetting can drag some gaseous fragments inside the bubble, leading to the formation of internal structures as confirmed by the examples shown in Fig. 16.

The fragmentation of the bubble, referred to as the “counterjet” by Lindau and Lauterborn (2003), typically occurs within a specific range of $\gamma$ values, ranging from 1.2 to 3, consistent with the range observed in this study. When $\gamma$ falls below 1.2, the bubbles collapse directly onto the surface during jetting, resulting in a bubble cloud ring that is visible in the holograms. Nevertheless, a bubble ejection similar to the counterjet has been observed for extremely low stand-off distances (Koch et al. 2021; Reuter and Ohl 2021) (e.g. $\gamma \lesssim 0.1$). In the latter case, the bubble fragments are produced by a pinch-off of the main cavity produced by an annular flow on the cavity cusp. A different situation is observed for $\gamma \gtrsim 3$ where the jetting is considerably weaker, leading to more spherical collapses. At larger stand-off distances, bubble rupture may still occur, but due to the improved shape stability of the cavities, the formation of satellite bubbles is less likely to occur. Additionally, as the nearly spherical bubble collapses generate a predominantly radial flow around the cavity, many of the detached fragments are quickly reabsorbed by the main bubble during re-expansion.

**Fig. 17:**
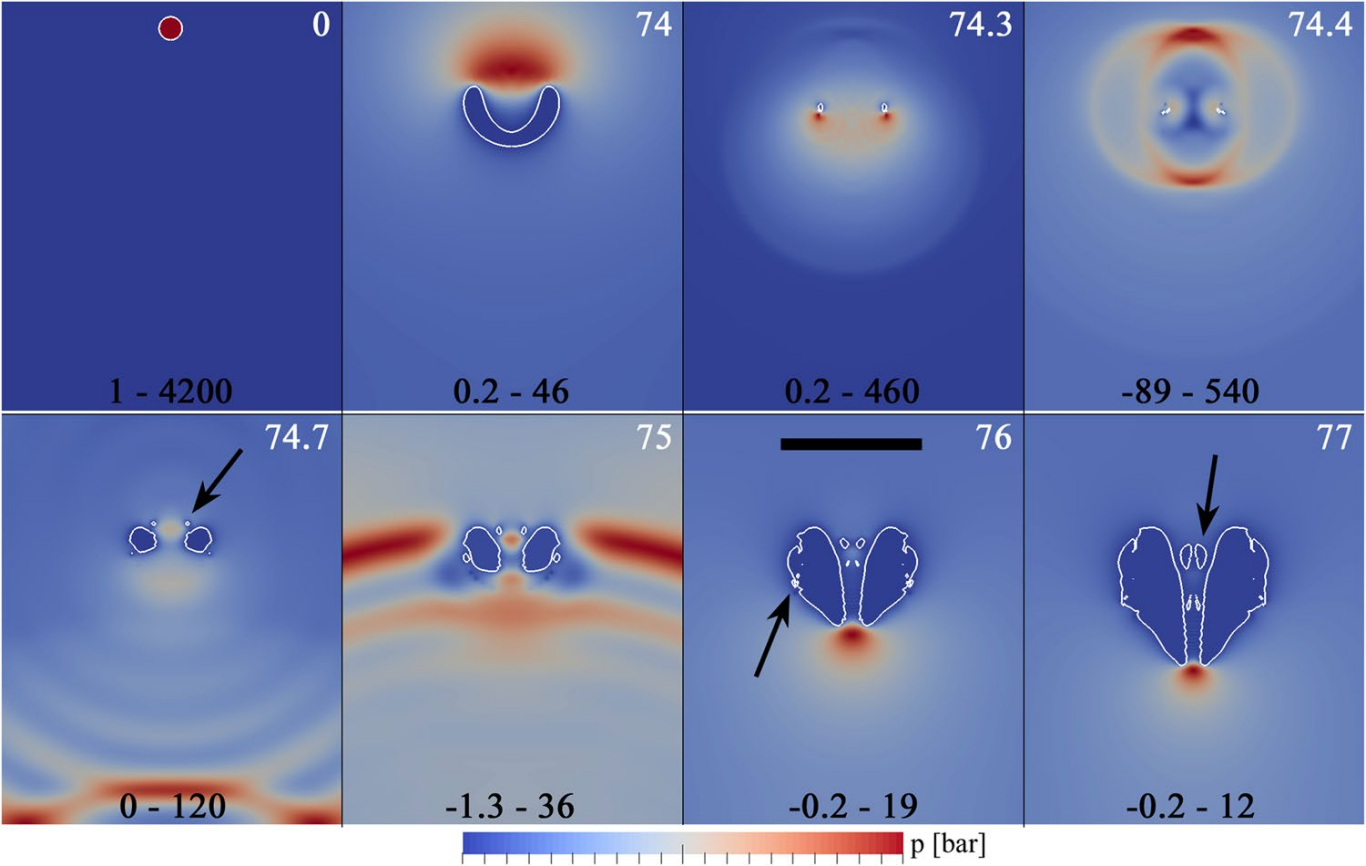
Simulation in axial symmetry for $\gamma = 1.77$ with $R_{\mathrm{{max}}} = 375\,{\upmu \text {m}}$ corresponding to the experimental case presented in Fig. 16. Snapshots of a cut through of the bubble (outlined by a white line) at various stages during collapse and rebound. The time in $\upmu {s}$ is indicated by the white numbers in the upper-right corner of the frames. The color represents the pressure in units of bar for the range shown in black numbers at the bottom of each frame. The solid boundary is located at the lower border of each frame (in white). The black scale bar represents $250\,{\upmu \text {m}}$. First row: The bubble seed is produced at $t=0$. During collapse a liquid jet forms, which is directed towards the solid. The second frame shows the bubble shortly before the jet impacts onto the opposite bubble wall. Third and fourth frame show the bubble shortly before and shortly after torus bubble collapse. Second row: rebound phase. Small bubble fragments are detached from the main cavity (pointed out by the arrows). Those are transported with the jet through the jet funnel, i.e. from $t=75\,\upmu {s}$ or reabsorbed by the bubble (outer fragment at $t=76\,\upmu {s}$). The fragments in the jet funnel expand over the course of time ($t=77\,\upmu {s}$)

**Fig. 20:**
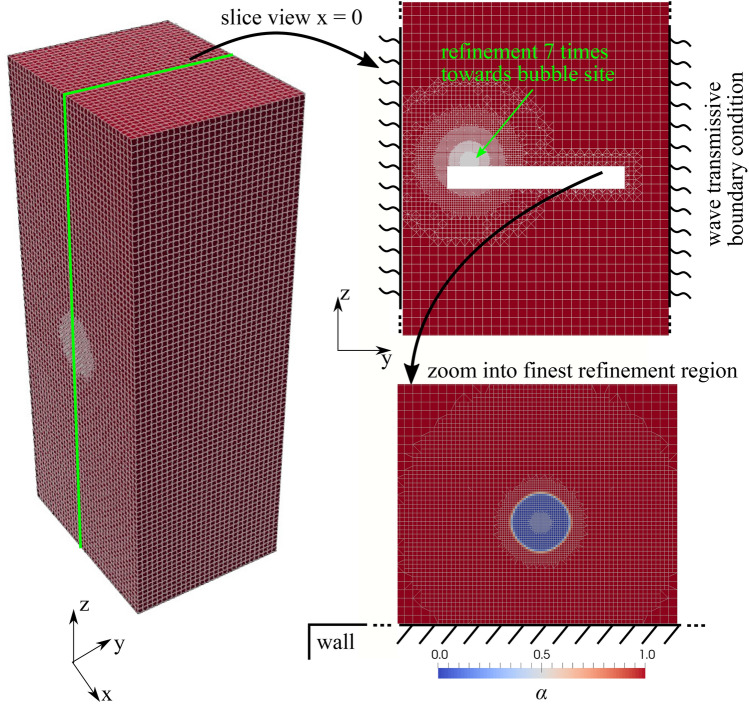
Mesh used for the numerical simulations. Red color indicates liquid (water) and blue color indicates gas (air), as represented by the volume fraction field $\alpha _l$. The green line indicates the cut at the plane where $y=0$. The right top frame shows the cut in that plane. The walls are taken to be approximately wave transmissive, mimicking the similar acoustic impedance of glass and water. The bottom right frame shows the mesh around the bubble generation site, close to the wall boundary of the cut-out cylindrical disc

The precise mechanism for the bubble fragmentation in the previous experimental examples was further investigated through the numerical simulations presented in Fig. 17. These simulations were performed with a minimum grid spacing of $1 \upmu$m in a region that completely covers the bubble. The numerical results confirm that the initial appearance of small fragments occurs immediately after the collapse, which is accompanied by powerful acoustic emissions reaching amplitudes of several hundreds of bar.

Such entrained and transported gas pockets might modify the stagnation pressure when impacting onto the solid (not shown in Fig. 16), and potentially they might collapse on their own then, contributing to cavitation damage. Further, it can be seen in the simulations and conjectured from the x-ray images that the gas pockets experience expansion during their transport in the jet flow. Impact and expansion both might lead to burst and thus contribute to ejection of very small droplets into the jetting bubbles. Such micro- or nano-sprays are supposed to be responsible for certain aspects of sonoluminescence and sonochemistry, namely the excitation and pyrolysis of non-volatile liquid components inside the bubble (Thiemann et al. 2017; Pflieger et al. 2019).

## Conclusion

In this study, we employed x-ray holographic imaging to investigate the jetting and internal structure of laser-induced bubbles collapsing near a solid surface. The bubbles were seeded off the symmetry axis of a circular glass plate, which resulted in non-axisymmetric collapse and jetting behaviour. The comparison and combination of experimental x-ray images with full 3D numerical simulations provide valuable insights into the phenomenon.

The advantages of x-ray holography over traditional optical imaging are evident. The absence of optical distortions caused by strong changes of the refractive index and the clear visualization of the gas cavity’s interior provided by x-ray imaging allow, for instance, for a realistic assessment of the jet’s shape and its speed, the tilting of its tip, and the Blake splash produced upon its impact on the solid surface (as shown in Fig. 4 and Fig. 5). All these critical features are accurately reproduced by the numerical model, demonstrating its reliability in capturing the complex dynamics of the system. This enabled us to extend beyond the experimental results and conduct a parametric analysis to investigate, for a fixed normalized plate displacement $\gamma =0.99$, the influence of the bubble seeding position ($\alpha _r$) on the curved shapes of the jets (see Fig. 10). The closer the bubble is seeded to the plate edge, the more the jet bends inward, i.e. towards the plate centre. The obliqueness of the jet is explained by the uneven inflow of the liquid surrounding the bubble during collapse. It is further observed from the simulations that the tilting of the jet leads to an impact on the solid with moving stagnation point, which is the location of momentary maximum pressure on the plate. This point moves from the impact position (further away from the plate edge) towards the projection point of the bubble centre (closer to the edge). Bending and stagnation point drift is stronger for bubbles seeded close to the plate edge (see Fig. 11).

Further interesting findings concern gas fragments entrained into the jet flow and thus into the rebounding bubble. This result could only be obtained with a clear view through the bubble, as provided by the x-rays (e.g. see Fig. 16). It could be relevant as well for cavitation erosion, but also for liquid injection mechanisms contributing to sonochemistry of non-volatile components. The details and connections with the counterjet phenomenon still remains to be investigated.

The results obtained in this study are representative of similar scenarios involving bubbles near the boundaries of irregular geometries with sharp edges. The implications of these findings extend beyond laser-induced cavities to bubbles generated by other methods and experimental scenarios such as acoustic nucleation in cleaning baths or cavitation from pressure gradients induced by a strong flow in turbo-machinery. Non-axisymmetric jetting scenarios are supposed to be generic and omnipresent in "real-world" situations, since a disturbance of symmetry is easily induced for many reasons. Thus, several features described in the present report should be representative for a considerably larger class of real-world systems. Among these phenomena are the non-straight and bent shapes of the jet, the entrainment of gas into the jet, the drifting stagnation points during jet impact, and non-uniform ring collapses. Implications of these effects are highly relevant for improving our understanding on cavitation erosion mechanisms; thus, they should be taken into consideration in the future research.

X-ray holography at an XFEL offers an unmatched capability to analyse rapid flow phenomena, such as those encountered in cavitation problems. This advanced technique proves invaluable in scenarios where optical access is hindered by distortions or opacity of the working liquid. The applicability of this methodology is promising for targeted applications in various industrial processes, enabling a deeper comprehension of bubble cleaning mechanisms, as well as the mitigation of cavitation erosion, paving the way for potential improvements in these areas. The effects of the boundary conditions in the jetting were not sufficiently considered in the literature and could be relevant for a better understanding of the processes that lead to cavitation erosion. Future research directly extending the present work could, for instance, explore a parameter space considering the curvature radius of the disc $R_D$ and its correlation with the impact angle of the jet mapping different values of $\gamma$ and $\alpha _r$. In a broader sense, the employed x-ray method can be applied to explore further situations of bubble–object or bubble–bubble interaction, but also problems of bubble nucleation and fast multiphase scenarios.

Note that for the purpose of the present paper, the interpretation of the X-ray micrographs in relation to jet and bubble geometry proved sufficient through a thorough comparison of near-field diffraction effects with simulations of wave-field propagation. Future work could be directed at a full inversion of the images by phase retrieval. To this end, smaller propagation distances in the parallel beam than in the current experiment would probably be useful. Furthermore, for projections at a single angle, phase retrieval only gives projected values, which then need to be interpreted based on geometric constraints. To this end, the fully 3D hydrodynamics simulations shown here could be extremely useful.

Aspects not yet fully exploited in the present work concern the reconstruction of matter density and shapes in three dimensions from the holographic data, and higher resolution than obtainable at optic wavelengths. Both requires additional steps of the methodology, but will be explored as well in the future. Moreover, x-ray imaging could possibly also be extended to full 3d by recording images from a series of rotation angles, or—simpler—by directing split beams onto the same scene for stereo-imaging.

### Supplementary Information

Below is the link to the electronic supplementary material.

Supplementary file 1 (avi 109 KB)

Supplementary file 2 (avi 53 KB)

## Figures and Tables

**Fig. 18:**
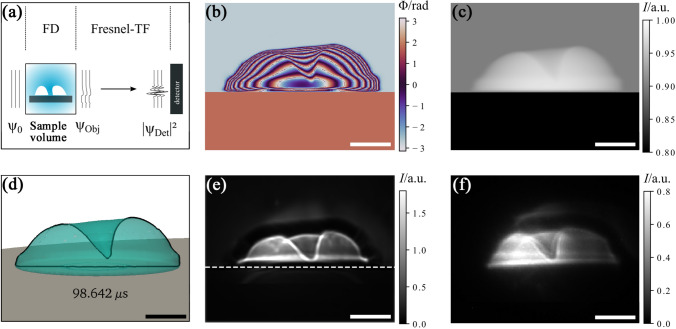
Overview of x-ray forward propagation simulations. **a** Schematic of the forward propagation steps, using a FD propagator through the sample volume and a Fresnel-TF propagator for free-space propagation to the detector. Panels **b** and **c** respectively depict the phase and intensity of the object wave function $\Psi _{Obj}$, directly behind the sample volume. Panel **d** shows a rendered outline of the simulated jetting bubble (green), with its cross section (black). **e** Simulated intensity $|\Psi _{Det}|^2$, i.e. the hologram in the detector plane after propagation of $\Psi _{Obj}$. The boundary of the glass plate is marked by the dotted line. **f** Measured hologram. Scale bars denote $200\,\upmu \hbox {m}$

**Fig. 19:**
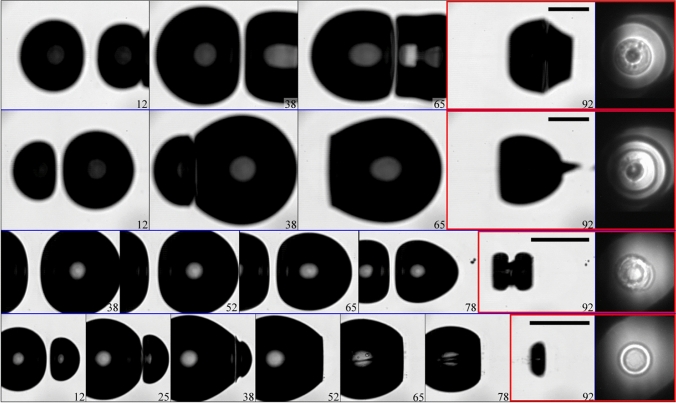
Internal structure of two bubbles with a different size jetting towards each other. The scale bar represents $500\,\upmu {m}$ and the numbers indicate time in $\upmu {s}$. The thin jet formed when the smaller bubble jets into the bigger one is visible in the last row of images. Here, the x-ray holographic images at the end of the sequence correspond to the last optical frame, marked in red

## Data Availability

The data that support the findings of this study are available from the corresponding author upon reasonable request. Data were recorded for the experiment at the European XFEL in October, 2019 and are available at DOI:10.22003/XFEL.EU-DATA-002544-00 (Hagemann et al. 2019).

## Appendix A: X-ray image processing and forward–propagation

A typical workflow for the image processing of x-ray phase-contrast holography would include a flat-field correction followed by numerical phase retrieval. Usually, this second step implies the reconstruction of the amplitude and phase of the sample’s image, which results in an image with a phase-contrast proportional to the electron density of the sample. This approach is conveniently used in synchrotron experiments (see, for example, Paganin et al. 2002; Bartels et al. 2015) and has also been demonstrated with single XFEL pulses (Hagemann et al. 2021; Vassholz et al. 2021). Reliable phase retrieval, however, requires a well working flat-field correction and sufficient pixel resolution on the acquired x-ray images, conditions which are not easy to meet for some setup configurations. An alternative method involves employing a parallel beam geometry to examine the raw detector images. Subsequently, a forward modelling approach can be utilized for an exemplary x-ray hologram, to describe typical features of the holographic imaging method. In this regard, future work could benefit from the use of smaller propagation distances in the parallel beam than in the current experiment. This would enable performing a full inversion of the images through phase retrieval.

In order to interpret the holograms we compare them with simulated holograms, based on data from our hydrodynamic simulations. In this scheme of educated fitting, we understand the origin of some characteristic and unintuitive features that occur in the x-ray holograms. The forward simulation of the x-ray holograms is based on a framework for numerical wave propagation that was previously used to simulate x-ray optics (Soltau et al. 2021). This approach is summarized in Fig. 18 and can be described in the following steps:

1. From the hydrodynamic simulations (Fig. 18d), we use the mass density of water $\rho (\vec r)$ and interpolate the data set on a Cartesian grid with a resolution of $2\,\upmu \hbox {m}$ and volume of 500 x 500 x 500 pixels. The Tait equation of state (Vassholz et al. 2021) is used to link between pressure and density (see Sect. 3.2). A map of the complex index of refraction $n(\vec r)$ for $E=$17.8 keV is calculated by scaling $n_{H_2O}$ (Schoonjans et al. 2011) according to $\rho (\vec r)$. Additionally, we insert a volume of quartz glass ($n_{SiO_2}$Schoonjans et al. 2011) to form the solid boundary that the bubble is jetting towards.
2. We propagate a plane wave through the sample volume using a finite differences (FD) propagator (Soltau et al. 2021; Fuhse and Salditt 2006). $n(\vec r)$ is passed to the propagator slice by slice. In this way, we calculate the complex object wave function $\Psi _{Obj}$ directly behind the sample (see Fig. 18b and c).
3. The wave function at the detector $\Psi _{det}$ is obtained by a single-step free-space propagation with a Fresnel contrast-transfer kernel (Soltau et al. 2021; Zhang et al. 2020; Voelz and Roggemann 2009). Here we use the geometry of the experiment, hence the effective Fresnel number $F_{pix} = 0.0071$ (additionally we used a correction factor of 1.2 to fine-tune the holographic regime). In this step, the interference effects develop which are characteristic for the holographic image. Before propagation, the image intensity is modulated by an effective illumination function, obtained from a measured hologram similar to the numerically calculated case (Fig. 18(f), for comparison with this exemplary measurement). This illumination function describes both the ellipsoidal aperture that results from clipping at the mirror through holes and the intensity distribution of the SASE pulse.
4. At the detector, we measure the intensity $|\Psi _{det}|^2$. To account for the point spread function of the imaging system, we perform a convolution with a Gaussian kernel with a FWHM of $13\,\upmu \hbox {m}$, corresponding to the width of 2 physical pixels.

We can now compare the simulated hologram (Fig. 18e) with a measured hologram (Fig. 18f) and a visualization of the corresponding hydrodynamic simulation (Fig. 18d). In this way, we can assess the level of realism of the hydrodynamic simulations to describe the structure of the jetting bubbles. As shown in this example, the holographic simulations were key to explain characteristic features found on the images. Some of these features are listed below:

i- A broad dark outer border and a bright inner edge can be seen at the boundary between bubble interior and water. This contrast is also visible but less pronounced at the boundary between bubble and jet. Note that the outline of the projected bubble corresponds to the outer edge of the dark border. This dark-to-bright contrast is typical for a sample with less density than its surrounding.
ii- The torus-like geometry of the upper part of the collapsing bubble leads to a downwards-curved bright arc.
iii- Close to the surface, the hologram shows a dark region that partially overlaps with some of the bubble features, like the Blake splash observed after the jet impact. This is due to both the bubble boundary (i) and the strong contrast of the edge of the glass boundary.
iv- In this example, the bubble forms a bell-like shape defined by an edge on the outer bubble boundary close to the surface, also referred to as “bubble pedestal”. This edge is also projected through the central region of the jetting bubble, visible as horizontal fringe and might lead to a slight deformation of the tip of the jet (see Fig. 18e).

Note that in the experiment the x-rays are partially coherent, and without a monochromator we assume a SASE bandwidth of $\approx 10^{-3}$, while the forward–propagation workflow is monochromatic. We hence attribute some differences between measured and simulated hologram to the difference in longitudinal coherence. For example, some regions of constructive interference are brighter in the simulation than in the measurement.

Due to the SASE process, each XFEL pulse is subject to random fluctuations of the spatial intensity pattern, as well as pulse energy and spectral structure. This leads to different and usually non-uniform illumination of the single-shot raw detector images. One can employ a flat-field correction that is based on principle component analysis of a set of empty images (Hagemann et al. 2021). In this scheme, for example, we determined the illumination function of the exemplary raw hologram shown in Fig. 18f and used it to modulate the forward-propagated hologram (Fig. 18e).

## Appendix B: X-ray images of bubble–bubble interactions

As an addition to the holographic images of bubbles jetting towards a solid boundary, Fig. 19 presents some examples of the ring structure produced when a bubble collapses in the vicinity of a second bubble. Here, the two cavities are induced (spuriously) by a single laser pulse along the axis of x-ray beam. In the measurements included in Fig. 19, the glass plate is not present or sufficiently far away from the bubbles not to play any role in their dynamics.

Interestingly, the direction of the jet is determined by the relative size of the cavities, i.e. the smaller bubble always collapses first producing a jet that pierces thought the second bubble. In the case where the bubbles have a similar size, the jetting occurs almost simultaneously and collide around the middle point between them, as shown in the third row of Fig. 19. On the other hand, in the cases where the dissimilarity in the bubble size is marked, the smaller bubble provokes a thin jet that goes through the bigger bubble inducing an annular vortex ring which forms a toroidal bubble, e.g. see the last row of Fig. 19. Apart from the last case, the x-ray images reveal a disturbed axial symmetry, not discernible from the video images. The undulated ring features and spikes apparently indicate some kind of splashing.

## Appendix C: Mesh of the numerical simulations

The mesh used for the numerical simulations is depicted in Fig. 20. It is initiated as a rectangular block with Cartesian cells of same edge length in each direction. Afterwards the regions are refined where the bubble is going to be generated and the region where the disc is going to be cut out. Around the bubble generation site 7 times concentric refinement is used. Around the region of the cylindrical disc, 3 times refinement is applied. Finally the disc is cut out of the mesh. The top of the disc coincides with $y=0$ and the amount of cells in *y*-direction is even, so that a flat top is guaranteed. The outer circle of refinement around the bubble generation site has a radius of 3.5 mm, the most inner circle has a radius of $30\,{\upmu \text {m}}$. In the OpenFOAM concepts, there are no hanging nodes at the refinement boundaries, but the cells are fit together via one layer of tetrahedral cells.
